# Insights into the Evolutionary Relationships of LytA Autolysin and Ply Pneumolysin-Like Genes in *Streptococcus pneumoniae* and Related Streptococci

**DOI:** 10.1093/gbe/evv178

**Published:** 2015-09-10

**Authors:** María Morales, Antonio J. Martín-Galiano, Mirian Domenech, Ernesto García

**Affiliations:** ^1^Departamento de Microbiología Molecular y Biología de las Infecciones, Centro de Investigaciones Biológicas, Consejo Superior de Investigaciones Científicas (CSIC), Madrid, Spain; ^2^Unidad de Genética Bacteriana, Centro de Investigación Biomédica en Red de Enfermedades Respiratorias (CIBERES), Instituto de Salud Carlos III (ISCIII), Madrid, Spain; ^3^Centro Nacional de Microbiología, ISCIII, Majadahonda, Madrid, Spain

**Keywords:** pneumococcus, main autolysin, pneumolysin, streptococci of the Mitis group, evolution, genomic island

## Abstract

*Streptococcus pneumoniae* (pneumococcus) is a major human pathogen. The main pneumococcal autolysin LytA and the pneumolysin Ply are two of the bacterium’s most important virulence factors. The *lytA*- and *ply*-related genes are also found in other streptococci of the Mitis group (SMG). The precise characteristics of the *lytA*-related—but not the *ply*-related—genes of SMG and their prophages have been previously described. A search of the more than 400 SMG genomic sequences available in public databases (ca. 300 for *S. pneumoniae*), showed *Streptococcus pseudopneumoniae* IS7493 to harbor four *ply*-related genes, two of which (*plyA* and *plyB*) have 98% identical nucleotides. The *plyA* homolog of *S. pseudopneumoniae* is conserved in all *S. pneumoniae* strains, and seems to be included in a pathogenicity island together with the *lytA* gene. However, only nonencapsulated *S. pneumoniae* strains possess a *plyB* gene, which is part of an integrative and conjugative element. Notably, the existence of a bacterial *lytA*-related gene in a genome is linked to the presence of *plyA* and vice versa. The present analysis also shows there are eight main types of *plyA−lytA* genomic islands. A possible stepwise scenario for the evolution of the *plyA−lytA* island in *S. pneumoniae* is proposed.

## Introduction

*Streptococcus pneumoniae* (pneumococcus) is a leading human pathogen that usually asymptomatically colonizes the mucosal surfaces of the upper respiratory tract in early childhood (Crook et al. 2004). Once carriage is established, however, *S. pneumoniae* may invade several sterile sites, leading to what is known as invasive pneumococcal disease. Indeed, the pneumococcus is responsible for episodes of community-acquired bacteremic pneumonia, bacteremia, and meningitis, mainly in children, the elderly, and immunocompromised patients (Henriques-Normark and Tuomanen 2013). Pneumococci are also a major cause of noninvasive diseases such as nonbacteraemic pneumonia, acute otitis media, sinusitis, and conjunctivitis. In addition to the capsular polysaccharide (CPS)—the main virulence factor of *S. pneumoniae*—many proteins are involved in the establishment and/or development of pneumococcal disease. These include the main autolytic enzyme LytA (a peptidoglycan hydrolase) and the pneumolysin Ply (a pore-forming toxin); both have been known for more than a century and are among the most widely studied proteins involved in pneumococcal virulence (Mitchell and Mitchell 2010; Ramos-Sevillano et al. 2015; Zahlten et al. 2015).

Pioneering work by Neufeld (1900) reported that pneumococci (but not other streptococci) rapidly “dissolve” in bile, a property still widely employed in the clinical setting (together with the optochin susceptibility test) for identifying *S. pneumoniae* among α-hemolytic, catalase-negative streptococcal isolates. Nowadays, however, sodium deoxycholate (Doc) is used in place of bile (Blaschke 2011). Doc-induced lysis is driven by the *N*-acetylmuramoyl-L-alanine amidase (NAM-amidase; EC 3.5.1.28) activity of the *lytA* gene product (García et al. 1986). LytA is also responsible for the characteristic autolytic behavior of the pneumococcus, the bacteriolysis caused by β-lactam antibiotics and, in cooperation with the glucosaminidase LytB, for the diplococcal morphology typical of this species (López and García 2004). LytA belongs to the amidase_2 family of proteins (whose members possess an *Amidase*_2 domain; PF01510), which includes Zn-dependent NAM-amidases and the peptidoglycan-recognition proteins (highly conserved pattern-recognition molecules of the immune system; Zhang et al. 2012). LytA was the first-discovered of the choline-binding family of proteins (López and García 2004). Choline-binding proteins are characterized by a choline-binding domain responsible for the binding of these proteins to the choline residues present in the teichoic and lipoteichoic acids of the bacterial surface (Sánchez-Puelles et al. 1990; Fernández-Tornero et al. 2001, 2002).

The *lytA* gene is located immediately downstream of the *lytR–cinA–recA–dinF* gene cluster in the *S. pneumoniae* genome (Mortier-Barrière et al. 1998). The same applies for the strain IS7493 of *Streptococcus pseudopneumoniae* (Shahinas et al. 2013), but not for *Streptococcus mitis* B6 (Denapaite et al. 2010) or *Streptococcus oralis* Uo5 (Reichmann et al. 2011) that lack a *lytA*-like gene at this position. Although the *lytA* gene has been considered exclusive to *S. pneumoniae* (Magomani et al. 2014), other, closely related streptococci (hereafter termed streptococci of the Mitis group [SMG]) and many pneumococcal and SMG prophages code for LytA-like lytic enzymes (Obregón et al. 2002; Romero et al. 2004, 2009; Llull et al. 2006). Fortunately, a number of characteristics allow for the accurate discrimination between typical pneumococcal *lytA* alleles (*lytA_Spn_*) and SMG (*lytA*_SMG_) or phage-encoded alleles (designated *lytA*_PPH_ or *lytA*_SPH,_ standing for pneumococcal and SMG prophages, respectively; this nomenclature follows that used previously by Morales et al. [2010]). These characteristics are: 1) *lytA_Spn_* alleles are 957 bp-long, whereas *lytA*_SMG_ (951 bp) shows characteristic signatures involving ≈100 nt positions and a distinctive 6-bp deletion (ACAGGC) between nucleotide positions 868 and 873. 2) In sharp contrast to LytA*_Spn_*, the LytA_SMG_ NAM-amidases of *S. pseudopneumoniae* and other SMG are inhibited by 1% Doc, which explains why these bacteria are not lysed in its presence—although they still lyse in the presence of 1% Triton X-100 (Díaz et al. 1992b; Obregón et al. 2002). 3) Even if all pneumococcal prophages reported to date code for a 318 amino acid-long NAM amidase (endolysin) (957-bp *lytA*_PPH_ alleles), there are significant nucleotide differences with respect to *lytA_Spn_* alleles, for example, ≥12 differences exist between the first 33 nucleotides of the prophage endolysin genes (*lytA*_PPH_ or *lytA*_SPH_) and the bacterial alleles *lytA_Spn_* and *lytA*_SMG_ (Llull et al. 2006). 4) All the endolysin-coding genes of phages—but none of the genuine bacterial *lytA* genes—are preceded by one (or two) holin/antiholin-like genes; this feature allows for easy discrimination between *lytA* genes of phage and bacterial origin. Interestingly, two temperate bacteriophages of *S. mitis*, ϕB6 and ϕHER, also code for LytA*_Spn_*-like endolysins with 318 amino acid residues (Romero et al. 2004), whereas the EJ-1 inducible prophage isolated from *S. mitis* strain 101 harbors a gene (*ejl*) with the 6-bp deletion characteristic of *lytA*_SMG_ alleles (Díaz et al. 1992a).

Rufus Cole first reported that pneumococci synthesize an intracellular hemotoxin, the action of which is prevented by small amounts of cholesterol (Cole 1914). In fact, *S. pneumoniae* synthesizes a cholesterol-dependent cytolysin or CDC (pneumolysin) that is an important virulence factor for this organism (Marriott et al. 2008; Dando et al. 2014). Ply induces cell death by pore formation and toxin-induced apoptosis (Mitchell and Dalziel 2014), although there is evidence showing that a nonhemolytic Ply allele may be associated with outbreaks of invasive disease (see below). Ply prevents complement deposition on *S. pneumoniae*, mainly by interfering with the classical pathway (Yuste et al. 2005).

Although the pneumolysin-coding gene (*ply*; 1,416 bp) is relatively well conserved among pneumococcal isolates (Marriott et al. 2008), Ply alleles showing reduced or nonhemolytic activity have been described (Kirkham et al. 2006; Jefferies, Johnston, et al. 2007; Jefferies et al. 2010; Harvey et al. 2011). Moreover, as stated above for *lytA*, *ply* orthologs have been described in some *S. mitis* strains (mitilysin; Mly) (Jefferies, Nieminen, et al. 2007) and most *S. pseudopneumoniae* isolates (pseudopneumolysin; pPly) (Johnston et al. 2010). Furthermore, a Ply-related, CDC cytolysin is synthesized by several *S. mitis* strains (Farrand et al. 2008). This protein has been named “lectinolysin” and is nearly identical to that previously designated as Sm-hPAF (standing for “*S. mitis*-derived human platelet aggregation factor”) (Ohkuni et al. 1997). This name, however, is misleading because “PAF” is the accepted standard abbreviation for “platelet-activating factor”—a glyceryl ether containing phosphoglyceride (Demopoulos et al. 1979). These proteins are secreted CDCs and contain a 36 amino acid-long, clevable signal peptide (SP). This is in sharp contrast to Ply that contains no SP (Mitchell and Dalziel 2014). Moreover, and unlike other previously characterized CDCs, lectinolysin-type proteins contain an additional domain of 162 amino acid residues (the *F5_F8_type C* domain; PF00754) that binds to fucose and difucosylated tetrasaccharides within Lewis y (Le^y^) and Lewis b (Le^b^) antigens. In the present study the term lectinolysin (Lly) is used, reflecting its ability to bind carbohydrate.

Recently, Denapaite et al. (2010) reported the *ply* gene to be separated from *lytA* by ≈10 kb in the genome of R6 (the common laboratory strain of *S. pneumoniae*), and that neither gene is present in the *S. mitis* B6 genome. Interestingly, these authors noted that, in the R6 strain, the *ply−lytA* region was flanked by a ≈100 bp direct repeat (87% identity between sequences); this is designated hereafter as *pl*REP (standing for *ply−lytA* REPeat). In contrast, only one copy of *pl*REP exists in the *S. mitis* B6 genome, and it overlaps the termination codon of the *dinF* gene (smi_1838) (Denapaite et al. 2010). Interestingly, polymerase chain reaction (PCR) experiments have shown that *ply*- and *lytA*-like genes are found in some SMG strains, and Southern blotting experiments have suggested that the presence/absence of one of these genes is somehow linked with the presence/absence of the other (Kilian et al. 2008). More recently, a putative genomic island that contains *pply* plus *lytA*_SMG_ has also been found in the *S. pseudopneumoniae* strain IS7493 (Shahinas et al. 2013), the only complete *S. pseudopneumoniae* genome reported to date. Taken together, these results suggest the existence of a *ply−lytA* island in pneumococci and some of its closely related SMG.

To get insight into the evolutionary relationships of *ply* and *lytA*, the genomic sequences (complete or not) of more than 400 SMG were examined. Novel data on *ply*-related genes were found. Taken as a whole, our analyses suggest that the *plyA* and *lytA* genes of *S. pneumoniae* may form part of a pathogenicity island.

## Materials and Methods

### Bioinformatic Genomic Analysis

The pneumococcal genomic sequences were aligned with the nucleotide sequences for the *ply*_D39_ (SPD_1726) and/or *lytA_Spn_*__D39_ (SPD_1737) genes of strain D39 (Acc. No. CP000410) (Lanie et al. 2007). The reason for selecting this strain for comparison lies in that the corresponding genes of the reference pneumococcal strains TIGR4 and R6 are also annotated in the same file. Alignments were performed using the BLAST (http://blast.ncbi.nlm.nih.gov/Blast.cgi?PAGE_TYPE=BlastSearch&BLAST_SPEC=MicrobialGenomes) or Clustal Omega (http://www.ebi.ac.uk/Tools/msa/clustalo/) (for multiple alignments) algorithms, employing the default parameters. The same programs were used to search for sequences similar to *pl*REP (see above). As previously reported for the R6 strain (Denapaite et al. 2010), two copies of this repeat were found in the D39 genome (or any other pneumococcal genome known to date; see below)*,* one located downstream of *ply* (coordinates 1720617−1720723) and the other overlapping the termination codon of *dinF* (coordinates 1730877−1730970). With the exceptions of strains NCTC 7465—the *S. pneumoniae* type strain—and GA02270, in which *ply, lytA*, and/or *pl*REP lie in two different contigs (and which unexpectedly were found to overlap for more than 100 identical nucleotides), only continuous genomic sequences including *ply*, *lytA* and the two *pl*REPs (241 strains) were analyzed further.

The description of the pneumococcal strains for which the genome was available in the National Center for Biotechnology Information (NCBI) BioProject database (http://www.ncbi.nlm.nih.gov/bioproject/, last accessed September 14, 2015) includes the sequence type (ST) (Enright and Spratt 1998) plus data such as serotype/serogroup. When this information was not available, the allele number and ST were searched for in the corresponding genomic sequences and assigned using the pneumococcal MLST website (http://pubmlst.org/spneumoniae/, last accessed September 14, 2015), which is hosted at the University of Oxford and has been funded by the Wellcome Trust. The serotype/serogroup was predicted by comparing the genomic sequences with those of capsular biosynthetic genes of *S. pneumoniae* (Bentley et al. 2006).

The ISfinder database (http://www-is.biotoul.fr, last accessed September 14, 2015) was searched for potential insertion sequences (ISs) (Siguier et al. 2006). For the prediction of protein function, the Pfam database (http://pfam.xfam.org/, last accessed September 14, 2015) was employed (Finn et al. 2014). The SignalP 4.1 server (http://www.cbs.dtu.dk/services/SignalP/, last accessed September 14, 2015) was used to predict SPs (Petersen et al. 2011).

## Results

The genomic sequences (complete or otherwise) of 1,466 streptococcal strains (representing at least 65 species) were obtained from the NCBI (http://www.ncbi.nlm.nih.gov/; last date accessed, October 25, 2014). Of these, 404 (27.5%) correspond to SMG (supplementary table S1, Supplementary Material online). The database also contains the genomes of 304 *S. pneumoniae* strains, 27 of which are complete (table 1). It should be noted that the quality of the assemblies has not been ascertained here and that there are different assembly levels ranging from short-read contigs to gapless, high-quality genomes.

**Table 1:** Summary of the *S. pneumoniae* Strain Material Examined

	Number
Genomes in databases^a^	304
Strains analyzed^b^	241
Serogroups^c^	21
STs	115^d^
PMEN clones	28 (169)^e^
*lytA_Spn_*/LytA*_Spn_* alleles	30/13^f^
*plyA_Spn_*/PlyA*_Spn_* alleles	35/10
*lytA_Spn_–plyA_Spn_* combinations	65

^a^Last date accessed, October 25, 2014.

^b^Of the 304 pneumococcal genomes available in databases, those analyzed were the ones in which both the *pl*REPs that flank *lytA* and *plyA* were located in the same contig.

^c^The nonencapsulated R6 strain is not taken into account here.

^d^Not including nine strains showing seven new and two unknown STs.

^e^The total number of strains belonging to PMEN clones, including SLVs and DLVs, are shown in parentheses.

^f^The *lytA*_30-_*_Spn_* allele was not translated into its protein since it may contain a sequencing error.

To properly analyze the structure and possible evolution of the *ply−lytA* island, we first examined the possibility that more than one *ply*-related gene may exist in some *S. pneumoniae* strains and related SMG and found several *ply*-related genes in SMG to be more frequent than previously thought. *S. pseudopneumoniae* IS7493 would appear to represent a special case since it codes for four Ply-related proteins.

### A Variety of *ply*-Like Genes Exist in Pneumococci and Other SMG

**Fig. 1.—:**
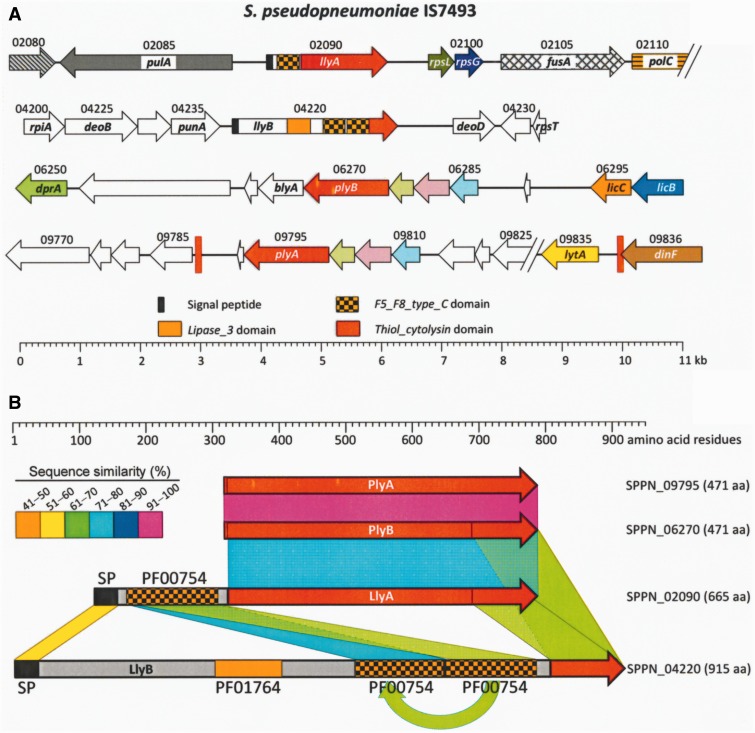
Pneumolysin genes and pneumolysin-like genes in the *S. pseudopneumoniae* IS7493 genome (Acc. No. CP002925) and their encoded proteins. (*A*) Pneumolysin-related genes are indicated by red arrows. Figures over the arrows correspond to SPPN_ loci. The locations of two direct repeats are indicated by vertical red bars. (*B*) Diagram of pneumolysin and pneumolysin-like proteins. The *Thiol_cytolysin* domain of PlyA/B and LlyA/B are shown in red. Amino acid sequence similarities are indicated by colors. SP, signal peptide; PF00754, *F5_F8_type_C* domain ; PF01764, *Lipase_3* domain.

Although apparently never mentioned before (Shahinas et al. 2013), the *S. pseudopneumoniae* IS7493 genome contains, in addition to the *pply* gene (SPPN_09795; 1,416 bp; *ply*_A hereafter), another gene named SPPN_06270 (*plyB* hereafter) that shares the same length and 98% of its nucleotides with SPPN_09795 (465 identical amino acid residues) (fig. 1). The *plyA* and *plyB* genes are located far apart in the chromosome. Notably, two more *S. pseudopneumoniae* genes are also *ply*-related: SPPN_02090 and SPPN_04220. The former (1,998 bp; *llyA*) is proposed to encode a thiol-activated cytolysin and corresponds to the Lly protein mentioned above, whereas the latter (2,748 bp) is annotated simply as coding for a hypothetical protein. The SPPN_04220 gene product (named LlyB hereafter) contains, as does LlyA, a SP and a *Thiol_cytolysin* domain (PF01289). Moreover, both proteins contain one (LlyA) or two (LlyB) *F5_F8_type_C* domains (see above). The latter protein also harbors a *Lipase_3* domain (PF01764) (fig. 1). The nucleotide sequence identity between either *llyA* or *llyB*, and *plyA*/*plyB* was ≤64%.

*Ply*-related genes similar to *llyA* or *llyB* also exist in various SMG, but apparently they are absent in pneumococci (supplementary figs. S1 and S2, Supplementary Material online). For example, a gene ≥98% identical to *llyA* is present in two *S. mitis* strains (SK597 and SK1080) and in the six *S. pseudopneumoniae* strains for which draft genomic sequences are available (supplementary fig. S1*A*, Supplementary Material online). The *llyA* gene is located immediately upstream of *pulA* and orientated in the opposite direction. Sequence comparisons also revealed the existence of other *llyA*-related genes—located downstream of the *parC* gene—in a variety of *S. mitis* strains (supplementary fig. S1*B*, Supplementary Material online). The products of these *llyA*-related genes were ≥90% identical to one another, whereas they showed 70−75% amino acid sequence similarity with the LlyA proteins mentioned above (data not shown). The terms LlyA1 and LlyA2 subfamilies of proteins will be used hereafter to designate those CDCs encoded by genes located, respectively, upstream of *pulA* or downstream of *parC*.

Additional *llyB-*like genes were found between *punA* and *deoD* in several SMG genomes (supplementary fig. S2, Supplementary Material online). Interestingly, most, if not all *S. pneumoniae* strains, contained a *llyB* remnant (or pseudogene)*.* In the case of the D39 strain, this remnant corresponds to genes SPD_0727 to SPD_0729, the latter being annotated as “hemolysin-related protein,” although apparently lacks cytolysin activity (Yamaguchi et al. 2009).

**Fig. 2.—:**
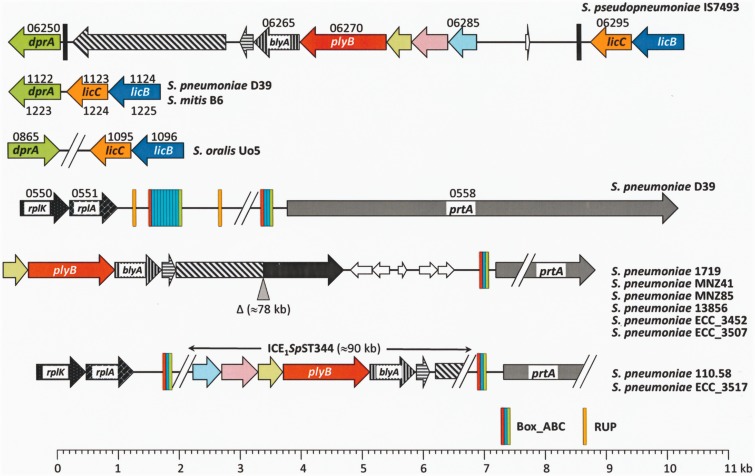
Diagram showing the chromosomal region containing (or not) a *plyB*-like gene in different SMG. The eight *S. pneumoniae* strains indicated at the bottom right of the figure are nonencapsulated. Thin arrows represent interrupted genes (pseudogenes). Genes sharing ≥90% identical nucleotides are represented by identical color and shading. The sequence types of the non-Ec-*Sp* strains shown are: 1719 and 13856, ST2996; MNZ41, ST6153; MNZ85 and ECC_3507, ST2315; ECC_3452 and 110.58, ST344; ECC_3517, ST1270. ST1270, ST2996, and ST6153 are SLVs of ST344.

The supernumerary *S. pseudopneumoniae plyB* gene is located immediately upstream of *blyA*, a gene coding for a 248 amino acid-long protein with an *Amidase_2* domain (as seen in the LytA NAM-amidase), and downstream of three genes (SPPN_06275−SPPN_06285) virtually identical to those located at an equivalent position in the *plyA−lytA* island (SPPN_09800, SPPN_09805, and SPPN_09810) (see below) (fig. 2). The *plyB* gene of *S. pseudopneumoniae* IS7493 resides in a 8.7 kb DNA fragment inserted between genes *dprA* and *licC* (coding for CTP:phosphocholine cytidylyl transferase) (Rock et al. 2001). These two genes are adjacent in the *S. pneumoniae* and *S. mitis* B6 genomes (but not in that of *S. oralis* Uo5) (fig. 2). It must be underlined that, in addition to *S. pseudopneumoniae* IS7493, a *plyB−blyA* tandem of genes exists only in a subset (group II) of nonencapsulated *S. pneumoniae (*non-Ec-*Sp*) strains (Keller et al. 2013; Valentino et al. 2014). Some non-Ec-*Sp* strains (group I) possess nonsense mutations in the CPS biosynthesis locus *cap*/*cps*. In contrast, group II non-Ec-*Sp* lack all the genes usually found in the *cap*/*cps* sequences of encapsulated *S. pneumoniae* isolates, but contains one or two *aliB*-like open reading frames (Hathaway et al. 2004). More recently, a new subset of group II non-Ec-*Sp* isolates containing a novel gene (named *nspA* or *pspK*) have been reported (Park et al. 2012; Salter et al. 2012). The group II non-Ec-*Sp* are related to a deep-branching classic lineage comprised mainly of the frequently identified ST344 and ST448 (Hilty et al. 2014). Interestingly, in pneumococci these genes are located in a chromosomal region (upstream of the *prtA* gene coding for a cell wall-associated serine protease [Bethe et al. 2001]) completely different to where they appear in *S. pseudopneumoniae* IS7493 (fig. 2). During the present work, a fully closed and annotated reference genome of a non-Ec-*Sp* isolate (strain 110.58; ST344) became available. Hilty et al. (2014) showed that ST344 non-Ec-*Sp* harbor two integrative conjugative elements (ICEs) not previously described in *S. pneumoniae*. A detailed analysis of the genome of strain 110.58 showed that *plyB* forms part of ICE_1_*Sp*ST344, and that this element may be partly deleted in other non-Ec-*Sp* pneumococcal isolates of the same (or different) ST (fig. 2).

In summary, our genomic screening showed the existence of a wide variety of *ply*-related genes with different chromosome locations in SGM species including *S. pneumoniae*.

### Some, but Not All, Mitis Group Streptococci Possess One Copy of the *plyA−lytA* Repeat

**Fig. 3.—:**
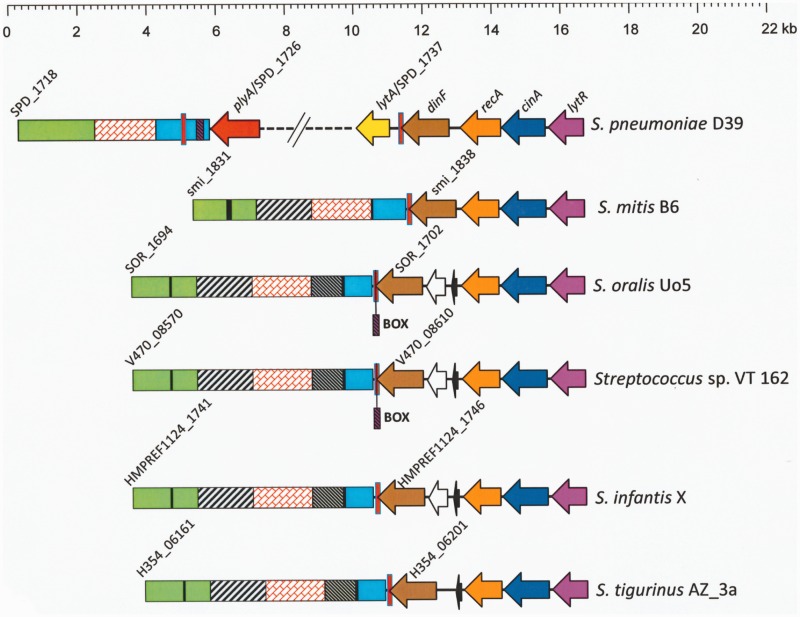
Diagram showing the gene organization around the *dinF* gene in selected SMG lacking the *lytA*_SMG_ gene. The region of the genome of *S. pneumoniae* D39 between SPD_1718 and SPD_1741 (*lytR*) is shown for comparison. Genes of known function are shown with arrows pointing in the direction of transcription. The vertical red bar represents the *pl*REP copy. Potential ISs are indicated by black bars. Regions showing ≥80% sequence similarity are represented by identical color and shading.

The nucleotide sequence of the *pl*REP copy overlapping the termination codon of the *dinF* gene in the *S. pneumoniae* D39 genome (nucleotide positions 1730877−1730970 in CP000410) was used as a query sequence to search the NCBI database of complete genomes (excluding *S. pneumoniae* genomes) for possible recombinational events. Only four genomes (all belonging to the *Streptococcus* genus) rendered significant similarities (*E* ≤ 10^−^^6^): *S. pseudopneumoniae* IS7493 (*E* = 10^−^^34^), *S. mitis* B6 (*E* = 10^−32^), *Streptococcus* sp. VT 162 (*E* = 10^−13^), and *S. oralis* Uo5 (*E* = 10^−11^). A similar search performed against the database containing streptococcal draft genomic sequences (also excluding *S. pneumoniae*) further confirmed that the existence (cutoff *E* value = 10^−6^) of at least one *pl*REP copy is restricted to some SMG: *S. pseudopneumoniae* (6 strains), *S. mitis* (30 strains)*, S. oralis* (12 strains)*, Streptococcus tigurinus* (4 strains)*, Streptococcus infantis* (6 strains), *Streptococcus dentisani* (2 strains)*,* and 21 out of 39 strains classified as *Streptococcus* sp. (supplementary table S1, Supplementary Material online). Interestingly, the presence of a *pI*REP copy at the 3′-end of *dinF* appears to be independent of the presence of a *lytA*_SMG_ gene in SMG (supplementary fig. S3, Supplementary Material online). The particular case of SMG with *lytA*_SMG_ genes, that is, all the sequenced strains of *S. pseudopneumoniae* and some *S. mitis*, is discussed below. All the examined strains showed well-conserved local synteny, and *pl*REP overlapped the termination codon of a *dinF*-like gene, in turn located immediately downstream of the *lytR−cinA−recA*-like gene cluster (fig. 3). In contrast, *dinF* appeared located far away from the *cinA−recA* genes (which typically lie together and in this order) in other streptococci also harboring a *dinF* homolog but lacking a sequence similar to *pl*REP (supplementary fig. S4, Supplementary Material online). The possession of a *lytR*-like gene in a position similar to that of *S. pneumoniae* appears to be uncommon in streptococci (not shown).

### A *plyA−lytA* Island Is Present in Most, If Not All, *S. pneumoniae* Isolates

**Fig. 4.—:**
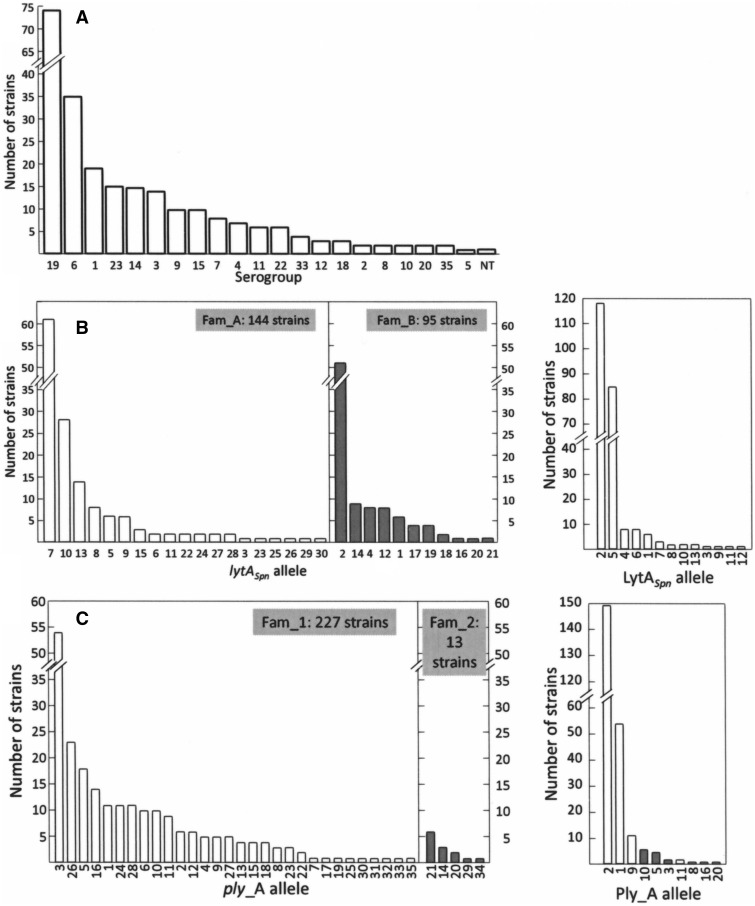
Serogroup, and *lytA_S__pn_*/*plyA* allele distribution, among the 241 *S. pneumoniae* strains analyzed. (*A*) Serogroups. Non-Ec, nonencapsulated. (*B*) Alleles of *lytA* (left) and LytA (right) in the sample. Open and gray bars indicate Fam_A and Fam_B *lytA* alleles, respectively. (*C*) Alleles of *plyA* (left) and PlyA (right). Open and gray bars indicate Fam_1 and Fam_2 *ply* alleles, respectively.

Table 1 summarizes some of the characteristics of the *S. pneumoniae* genomes analyzed in this study. Of the 304 genomic projects found, 241 genomic sequences (corresponding to the 21 serogroups most frequently found in clinical specimens [Grabenstein and Musey 2014]; fig. 4*A*) fulfilled the requirement mentioned above, that is, to have two copies of the *pl*REP element flanking the *ply* and *lytA* genes in one contig (or in two unnoticed overlapping contigs) (supplementary table S2, Supplementary Material online). It should be noted that the 63 pneumococcal strains that did not meet the above criterion did contain, nonetheless, a copy of both *ply* and *lytA_Spn_* genes (supplementary table S3, Supplementary Material online). In every case, the location of the *ply* gene corresponded to that designated as *plyA* above (fig. 1).

Nearly half of the isolates examined differed in genotype (115 STs were recognized in the present study) (table 1). Further, up to 70% of the strains belonged to one of 28 (out of the possible 43) Pneumococcal Molecular Epidemiology Network (PMEN) clones. PMEN clones are resistant to one or more antibiotics in wide clinical use and dominate the population of antibiotic-resistant pneumococci. Globally susceptible clones known to be important in disease are also included in the PMEN clone data set (http://spneumoniae.mlst.net/pmen/pmen.asp, last accessed September 14, 2015).

Thirty different *lytA_Spn_* alleles were found (fig. 4*B* and supplementary table S4, Supplementary Material online). The deduced amino acid sequences were then aligned, resulting in the identification of 13 different NAM-amidases. Similarly, 35 *plyA* alleles (coding for 10 PlyA cytolysins including a new allele given the preliminary designation “allele 20”; see below) were found (fig. 4*C* and supplementary table S5, Supplementary Material online). The *lytA_Spn_* alleles were classified into two families (Fam_A and Fam_B) according to a previous proposal (Morales et al. 2010). This classification resulted from the finding that these two families of pneumococcal *lytA_Spn_* alleles (but not the corresponding NAM-amidases) can be differentiated by PCR and digestion with restriction enzymes on the basis of a highly polymorphic region located around position 453 of *lytA_Spn_* (taking the first nucleotide of the ATG initiation codon as position 1). It is noteworthy that alleles LytA_2__*_Spn_* and LytA_5__*_Spn_* together represented more than 85% of the NAM-amidases encoded by the pneumococcal strains studied in the present work (fig. 4*B*). Similarly, we propose a subdivision of *plyA* alleles: Fam_1 clustered alleles with a full length gene (1,416 bp), while the 1,410 bp alleles—which lack the nucleotides located between positions 808 and 813 (GTCAAC) encoding Val_270_ and Lys_271_—formed Fam_2 (fig. 4*C*). Since previous studies had assigned numbers (1 through 19) to the different PlyA alleles found (but not to the corresponding genes) (Jefferies, Johnston, et al. 2007; Jefferies et al. 2010; Harvey et al. 2011), the *plyA* alleles were renumbered, but the nomenclature used for their encoded proteins was maintained.

The serotype 3 SPNA45 strain (ST6934) (supplementary table S2, Supplementary Material online) appears to represent an unusual case among pneumococci in its having undergone a lengthy deletion extending from the 5′-region of *plyA* to the 3′-region of *lytA*. The origin of the SPNA45 strain is obscure although it may correspond to a serotype 3 isolate of equine origin. These equine isolates lack hemolytic activity and are Doc-insoluble (Whatmore et al. 1999). Notably, a comparison between the genomes of SPNA45 and D39 revealed that, in addition to the *ply−lytA* deletion, two large rearrangements occurred in the former strain (supplementary fig. S5, Supplementary Material online).

### Gene Organization of the Pneumococcal *plyA−lytA* Island

**Fig. 5.—:**
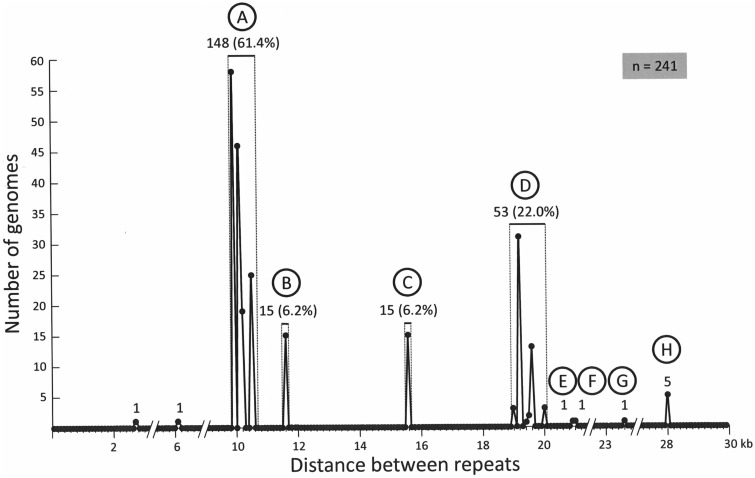
Size distribution of the *plyA−lytA* island in 241 *S. pneumoniae* strains. Letters A−H correspond to the arrangements shown in figure 6.

The length of the *plyA−lytA* island differed greatly among *S. pneumoniae* strains (supplementary table S2, Supplementary Material online). When the distance separating the flanking *pl*REP was approximated to its nearest value, a bimodal distribution with peaks at ≈10 kb and ≈19 kb (together accounting for >80% of strains) was observed (fig. 5). A clear correlation was seen between length and clonal origin: the 15 strains with an 11.6 kb-long island belonged to the clonal complex formed by the PMEN global clone North Carolina^6A^-23 (ST376) and several of its double locus variants (DLVs) (ST1296, ST1339, ST2268, ST8207), whereas those with a 15.6-kb-long island belonged to the complex formed by the PMEN clone Spain^9V^-3 (ST156) and three of its single locus variants (SLVs) (ST3148, ST4026, ST4464). In addition, the five strains with the longest island (28.2 kb) were seen to be members of the clonal complex formed by the PMEN clone Maryland^6B^-17 (ST384) and its SLV, ST2150 (supplementary table S2, Supplementary Material online).

**Fig. 6.—:**
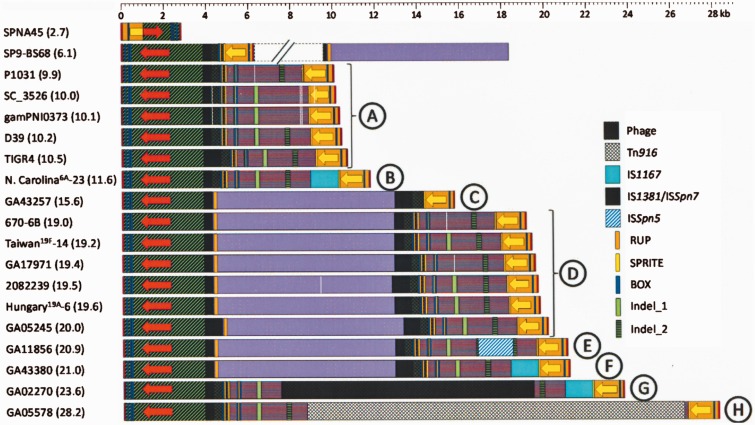
Organization of the various pneumococcal *plyA−lytA* islands. The name of one representative strain of each class is shown at the left. Red and yellow arrows correspond to *plyA* and *lytA* genes, respectively. The arrows show the direction of transcription. Regions with ≥85% sequence identity are represented by identical color and shading.

Figure 6 shows a diagram of the region encompassing the *pl*REPs in pneumococcal genomes. Leaving out SPNA45 and SP9-BS68, eight different genomic arrangements (designated A to H) were recognized. These were clustered into two major groups. The most numerous (169 out of 241 strains; 70%) was named Gr1 and includes arrangement types A (9.9−10.5 kb), B (11.6 kb), G (23.6 kb), and H (28.2 kb); the other (Gr2) comprises types C−F. The main differences among arrangements of the same group reside in the presence/absence of different ISs (either complete or partly deleted), some insertions/deletions (indels), and/or repeat sequences (fig. 6). To be precise, the ISs present/absent were IS*1167*, IS*1381* (also named IS*Spn7*), and IS*Spn5*. Moreover, at least one copy of the repeat sequences BOX, RUP, and SPRITE, characteristic of pneumococci (Croucher et al. 2011), was also present.

The main difference seen between Gr1 and Gr2 strains resided in that the latter possesses an additional ≈8.6-kb DNA fragment (fig. 6). By way of example, this fragment is located between nucleotide positions 1,872,219 and 1,880,825 of the *S. pneumoniae* strain 670-6B genome (Acc. No. CP002176). The same fragment was identified in strain SP9-BS68, but is located in a contig different to that containing the *plyA−lytA* island. This 8.6-kb DNA fragment appears to be the result of a horizontal transfer event from an unknown source. In fact, the maximum closeness (60−70% identity) between this DNA fragment and any other sequence in the databases (last date accessed, October 25, 2014) corresponds to a fragment in the chromosome *of S. suis* D9, but apparently not in other *S. suis* isolates (supplementary fig. S6, Supplementary Material online). Moreover, sequence comparisons suggested that this DNA fragment codes for proteins that are annotated as involved in transport and related functions (supplementary fig. S6, Supplementary Material online).

The *S. pneumoniae s*train GA02270 (fig. 6), which was found to have a 23.6-kb island (arrangement G), is a DLV (ST1339) of the North Carolina^6A^-23 PMEN clone. In contrast, some members of this clone possess a shorter island (11.6 kb; arrangement B). Strain GA02270 appears to be unique in that it has integrated a potentially defective prophage (≈ 12 kb) into its type B *plyA−lytA* island (supplementary fig. S7, Supplementary Material online). In a similar manner, the strains with a type H arrangement (28.2 kb) differ from type A strains by the insertion of a copy of Tn*916* (≈18 kb) (fig. 6). This mobile genetic element is widely distributed among important human pathogens, including *Enterococcus faecalis*, *Clostridium difficile*, *Staphylococcus aureus,* and *S. pneumoniae*, and it harbors the tetracycline resistance-conferring *tet*(M) gene (Santoro et al. 2014).

A search for a possible genetic link between particular *plyA* and *lytA* alleles among different arrangements gave no evidence of association. In other words, no preference for association between *lytA* alleles belonging to families A or B and *plyA* alleles of Fam_1 or Fam_2 was seen (data not shown). Nevertheless, as many of the alleles may encode identical proteins (or different proteins with similar enzymatic activity) (supplementary tables S4 and S5, Supplementary Material online), the existence of a functional association between PlyA and LytA proteins in different arrangements could not be completely discarded. Data relative to the specific activity of these proteins only exist in the case of PlyA. Actually, the *S. pneumoniae* strains studied here can be grouped into four categories on the basis of PlyA alleles (Jefferies, Johnston, et al. 2007): 1) alleles 1, 2, 9, and 11, with a specific activity of about 4 × 10^5^ hemolytic units (HU) mg^−1^; 2) alleles 8 and 10, with slightly reduced hemolytic activity (≈10^5^ HU mg^−1^); 3) allele 3, having a very reduced hemolytic activity (≈3−7 × 10^3^ HU mg^−1^), and 4) allele 5 that completely lacks hemolytic activity (supplementary table S5, Supplementary Material online). It should be noted that, all together, alleles 1, 2, 9, and 11 were present in near 90% of the studied strains (215 out of 240; fig. 4*C*). As the enzymatic activity of the LytA alleles had not been previously determined, we decided to determine the activity of encoded NAM-amidases. The *lytA* genes coding for LytA alleles 1, 2, 4, 5, or 7 were cloned into the pT7-7 expression vector, overexpressed and the corresponding NAM-amidases were purified (Supplementary Material online). These four alleles correspond to approximately 90% of the studied strains (220 strains out of 239; fig. 4*B*). Only LytA_7 showed a slight (albeit statistically significant) increase in the specific activity of the NAM-amidase (supplementary table S4, Supplementary Material online). Unfortunately, and spite of our efforts, attempts to find any significant functional association between PlyA and LytA alleles of high/low specific activities were unsuccessful (data not shown).

As mentioned above, Ply contains no SP and autolysis caused mainly by the LytA NAM-amidase has long been recognized for its ability to rapidly release the majority of the pneumococcal hemolytic activity (Mitchell and Dalziel 2014). Early results had shown that LytA was required for optimal biofilm formation in vitro (Moscoso et al. 2006). A similar requirement for Ply has also been reported recently (Shak et al. 2013). Interestingly, the role of Ply on in vitro biofilm formation appears to be independent of its hemolytic activity, as pneumococcal strains synthesizing a nonhemolytic Ply allele (allele 5; see above) were still able to form biofilms (Shak et al. 2013). We have now confirmed that both proteins (Ply and LytA) are important for optimal biofilm formation in *S. pneumoniae*, although the biofilm forming capacity of a double *ply lytA* mutant was not significantly different to that of the single *ply* mutant (supplementary fig. S8, Supplementary Material online).

### A *plyA−lytA* Island Is Also Present in Several SMG Different to *S. pneumoniae*

**Fig. 7.—:**
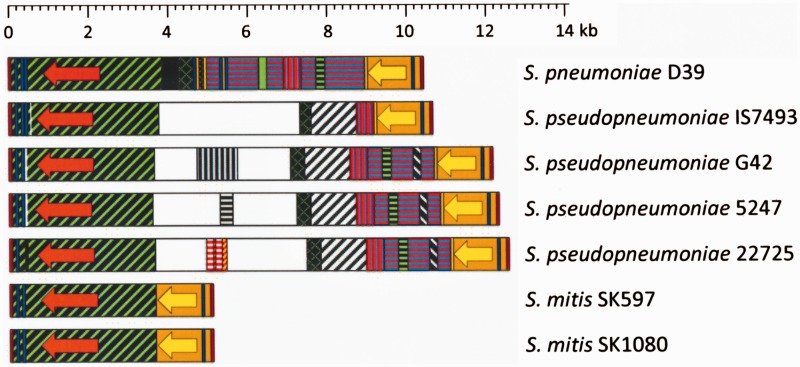
Diagram showing the *plyA−lytA* islands in two species of SMG. The *S. pneumoniae* D39 strain is shown for comparison. Red and yellow arrows correspond to the *plyA* and *lytA* genes, respectively. The arrows indicate the direction of transcription. Regions having ≥85% sequence identity are represented by identical color and shading.

As mentioned above, all the *S. pseudopneumoniae* strains whose genome is known in either full (1 strain) (Shahinas et al. 2013) or draft (6 strains) version (this study), appear to code for a LytA_SMG_ NAM-amidase. However, we have now found that only a minority of *S. mitis* strains (3 strains out of 31) contained *bona fide* homologs of this gene. All these strains were found to contain a *plyA* homolog (nucleotide similarity ≥96%), although only four *S. pseudopneumoniae* and two *S. mitis* strains could be examined in detail since only these contained the *plyA* and *lytA*_SMG_ genes in the same contig (fig. 7). These results fully confirmed the previous proposal that the presence/absence of one of these genes is somehow linked with the presence/absence of the other (Kilian et al. 2008). *S. pseudopneumoniae* strains showed a *plyA−lytA* island arrangement more complex than that of *S. mitis* strains, although in both species the island was always flanked by *pl*REP, as in *S. pneumoniae*. The intervening sequences found in *S. pseudopneumoniae* potentially encode proteins of unknown function (not shown).

### A Plausible Scenario for the Evolution of the *plyA−lytA* Island in *S. pneumoniae*

**Fig. 8.—:**
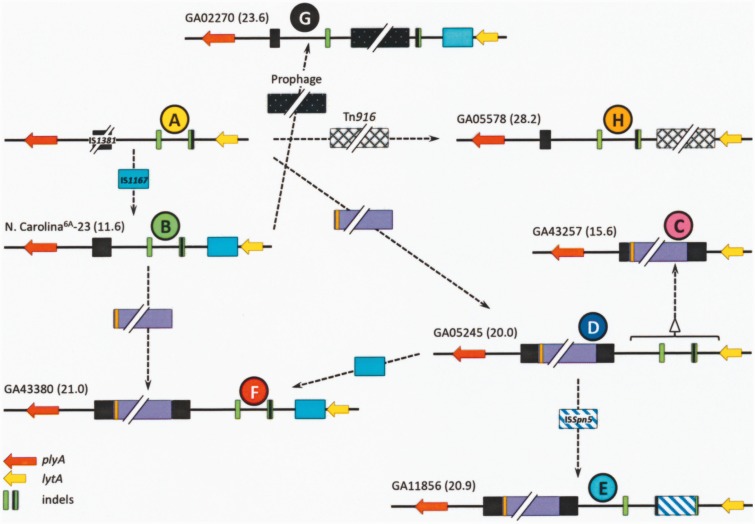
Possible evolutionary steps leading to the present diversity of the *plyA–lytA* island of *S. pneumoniae*. It is proposed that the starting point corresponds to an island of about 10 kb that was introduced into the pneumococcal genome from an unknown source (A). Arrangements B, G, and H may arise by insertions of IS*1167* (B), a prophage (G), and/or Tn*916* (H), respectively. Insertion of an ≈8.6 kb fragment (mauve bar) and successive insertions/deletions (indels) are proposed to have taken place for arrangements C−F.

Arrangement A (ca. 10 kb-long) was assumed to represent the founder since it is by far the most widely distributed among otherwise distantly related *S. pneumoniae* strains, including nonencapsulated isolates (deduced using a phylogenetic tree of the core genome of 44 *S. pneumoniae* genomes [Donati et al. 2010], and a dendrogram based on genomic BLAST of 320 pneumococcal strains [supplementary fig. S9, Supplementary Material online]). In contrast, arrangement D appears to have arisen and/or horizontally transferred independently on several occasions. Based on these data, Figure 8 provides a possible evolutionary pathway compatible with the observed diversity of the pneumococcal *plyA−lytA* island. The sources of the proposed earliest island, and of the ≈8.6-kb fragment characteristic of arrangements C−F, are unknown. Additional indel events explain the formation of the rest of the island arrangements. Similar, albeit apparently independent, events may have occurred in *S. pseudopneumoniae* strains (fig. 7). However, the uncommon *S. mitis* strains with a *plyA−lytA* island (namely, strains SK597, 1080, and likely, SK564), were seen to possess an arrangement closely related to that of the *S. pneumoniae* strain SP9-BS68 (fig. 6) that may have arisen through additional genomic rearrangements.

## Discussion

Although all the *S. pneumoniae* strains analyzed here contain one *plyA* copy, which forms an island with the *lytA_Spn_* gene, most if not all of the group II non-Ec-*Sp* strains also harbor a supernumerary *plyB* gene elsewhere in their genome as part of an ICE element (ICE_1_*Sp*ST344) (fig. 2). While strains that lack a capsule have substantially reduced virulence in invasive infections, non-Ec-*Sp*—which are unaffected by current vaccines that target the pneumococcal CPS —cause up to one-third of all episodes of acute conjunctivitis outbreaks (Martin et al. 2003; Marimon et al. 2013). The reasons underlying the conjunctival tropism of NT pneumococci are not completely understood (Valentino et al. 2014). However, due to the presence of a *plyB* copy in their genomes, non-Ec-*Sp* may synthesize more Ply than encapsulated isolates. It should be remarked that the region containing the *plyA* promoter (Walker et al. 1987) is identical to that of *plyB.* Interestingly, the PlyB pneumolysin of the non-Ec-*Sp* 110.58 strain (and other non-Ec-*Sp*) is identical to allele 7 of Ply_A that displays full hemolytic activity (Jefferies, Johnston, et al. 2007) (data not shown). Experimental evidence exists to suggest that Ply-deficient bacteria show greatly reduced virulence in animal models of ocular keratitis or endophtalmitis, and that immunization with Ply showed a protective effect (Mitchell and Dalziel 2014). To the best of our knowledge, however, no study has reported either an increased production of Ply in non-Ec-*Spn* or that this may be the reason for a greater capacity to cause conjunctivitis.

According to the present data, genes coding for Lly-related proteins are quite common in *S. mitis*, uncommon in *S. pseudopneumoniae*, and either absent (this work) or, perhaps, seldom present (Farrand et al. 2008) in *S. pneumoniae*. Only a gene remnant potentially encoding a truncated protein of the LlyB type appears to be present in pneumococcal genomes (supplementary fig. S2, Supplementary Material online). It is unclear whether the extant pneumococcal pseudogene has resulted from the accumulation of mutations, or whether a gain-of-function mechanism resulted in the presence of a complete open reading frame in some SMG. Actually, only four mutations (two single nucleotide insertions plus two transition mutations) are required to convert the SpnNT_01477−01479 remnant (supplementary fig. S2, Supplementary Material online) into a potential gene coding for a 771-long Lly_B-like polypeptide in the non-Ec-*Sp* strain 110.58 (data not shown).

We have also found that the *S. mitis* strain SK597, which appears to be a mitis/oralis hybrid (Kilian et al. 2008), harbors genes encoding Lly-like proteins of the LlyA1, LlyA2, and Lly_B type (see above). It is tempting to speculate that harboring several Lly homologs might represent an advantage for virulence as the different proteins may be better adapted to particular cell types or surface receptors. *S. mitis* proteins of the LlyA family, and particularly those of the LlyA1 subfamily, have been shown to bind cholesterol and the human glycophosphatidylinositol-anchored CD59 (Kimberley et al. 2007) prior to triggering pore-formation (Tabata et al. 2014). The relative redundancy of *lly*-like genes observed in *S. mitis* is intriguing. CDCs are part of the membrane attack complex/perforin (MACPF)/CDC superfamily of pore-forming proteins that include the MACPF family (Gilbert 2014). For a long time it was believed that cholesterol served as the membrane receptor for CDC toxin monomers (Gilbert 2010). However, although it is known that Ply requires membrane cholesterol for its cytolytic effects, it has not been definitively shown that cholesterol functions as the cellular receptor. Further, Lly-type proteins possess an *F5_F8_type_C* lectin-like domain that binds to fucose and to the difucosylated tetrasaccharides Le^b^ and Le^y^ (Farrand et al. 2008; Feil et al. 2012), which would increase the number and types of binding receptors in the target cells. Quite unexpectedly, very recent results have revealed that Ply and streptolysin O (from *Streptococcus pyogenes*) also have lectin activity and bind glycans (Shewell et al. 2014).

The tendency of the pneumococcal *lytA* and *plyA* genes to persist recurrently in relative vicinity observed in this study suggests that their corresponding products might function together, forming a (patho)physiological protein network. The clustering of genes in bacteria contributes to the attainment of high local protein concentrations close to their encoding genes (because transcription and translation are simultaneous); thus, they may selectively interact even when they are not cotranscribed (Lawrence 1999; Montero Llopis et al. 2010). Previous experiments have shown that *plyA* and *lytA* are required for optimal biofilm formation in vitro (Moscoso et al. 2006; Shak et al. 2013). However, we have now found that a double *ply lytA* mutant did not show any additional impairment on biofilm-forming capacity, as compared with the single mutants (supplementary fig. S8, Supplementary Material online). A possible explanation may be that—in contrast with what has been a working hypothesis for many years, that is, that Ply escapes from the cell on lysis caused by the pneumococcal LytA NAM-amidase (Marriott et al. 2008)—near 50% of the Ply is localized to the cell wall compartment in a LytA-independent manner (Price and Camilli 2009).

The LuxS/autoinducer 2 quorum-sensing system is known to regulate the transcript levels of both *lytA* and *ply* genes during early biofilm formation (Vidal et al. 2011). Moreover, *lytA* and *ply* have been reported to show similar levels of expression in nasopharyngeal samples taken from colonized (but otherwise healthy) children (Sakai et al. 2013). Additional support for the coordinated expression of *ply* and *lytA* being important in disease development comes from observations made in mixed infections of influenza A virus (IAV) and *S. pneumoniae*. Mechanisms of pathogenesis include IAV destruction of the respiratory epithelial cells with subsequent impairment of mucociliary bacterial clearance, upregulation or exposure of receptors for pneumococcal adhesion, and IAV-induced suppression of innate and adaptive immune responses to *S. pneumoniae* (McCullers 2014). It has recently been reported that infection with IAV induces the release of bacteria from biofilms. These dispersed bacteria show differential virulence gene expression that results in a significantly increased ability to disseminate and cause invasive infections (Marks et al. 2013). Compared with biofilm-grown bacteria, *lytA* and *ply* are upregulated in IAV-dispersed cells (Pettigrew et al. 2014). In agreement with early results (Canvin et al. 1995), recent experimental evidence from a murine model of infection shows coordinated activity of Ply and LytA to be important in complement evasion and in the establishment of pneumococcal pneumonia and sepsis (Ramos-Sevillano et al. 2015).

The CPS is a critical virulence factor of *S. pneumoniae* and is generally assumed to distinguish pathogenic pneumococci from commensal SGM by its capacity to avoid phagocytosis. Most (but not all) SMG synthesize neither CPS nor other pneumococcal virulence factors (e.g., IgA1 protease) (Kilian et al. 2008). In a similar way, the present study has revealed that a *plyA–lytA* island is missing in ≈90% of *S. mitis* and completely absent in other SMG, with the only exception of *S. pseudopneumoniae* isolates that consistently harbor such an island (fig. 7). It should be noted, however, that the *lytA*_SMG_ alleles encode a NAM-amidases with reduced specific activity, that is*,* ≈50% of that LytA*_Spn_* (Obregón et al. 2002). Therefore, our results give further support to the hypothesis that commensal streptococci, for example, SMG, gradually evolved from the pathogen *S. pneumoniae* by genome reduction (Kilian et al. 2008, 2014).

Compared with the core genome, genomic islands frequently have high percentage of genes coding for proteins with unknown function (Hsiao et al. 2005), and in particular, pathogenicity islands have several additional characteristics such as presence of genes encoding virulence factors, mobility genes (integrases, transposases), phage-related genes, direct repeats, and insertion sequences, that allow their potential identification in bacterial genomes (Che et al. 2014). The results presented here reveal that most of these features are also present in the *plyA–lytA* region and support the proposal that the *plyA* and *lytA* genes of *S. pneumoniae* may form part of a pathogenicity island, although further experimental and/or epidemiological work would be required to establish a real link with pathogenesis.

## Supplementary Material

Supplementary figures S1−S8 and tables S1−S5 are available at *Genome Biology and Evolution* online (http://www.gbe.oxfordjournals.org/).

Supplementary Data
