# Supplementary material for: Insights into the Evolutionary Relationships of LytA Autolysin and Ply Pneumolysin-Like Genes in Streptococcus pneumoniae and Related Streptococci
Source: Genome Biol Evol. 2015 Sep 10;7(9):2747–61. doi: 10.1093/gbe/evv178 (PMC4607534; doi:10.1093/gbe/evv178)
Supplement: Supplementary Data [file supp_7_9_2747__index.html]

Insights into the Evolutionary Relationships of LytA Autolysin and Ply Pneumolysin-Like Genes in Streptococcus pneumoniae and Related Streptococci — Supplementary Data 

# Insights into the Evolutionary Relationships of LytA Autolysin and Ply Pneumolysin-Like Genes in *Streptococcus pneumoniae* and Related Streptococci

## Supplementary Data

files

- Supplementary Data - docx file
- Supplementary Data - xlsx file
- Supplementary Data - xlsx file
