# Supplementary material for: Insights into the Evolutionary Relationships of LytA Autolysin and Ply Pneumolysin-Like Genes in Streptococcus pneumoniae and Related Streptococci
Source: Genome Biol Evol. 2015 Sep 10;7(9):2747–61. doi: 10.1093/gbe/evv178 (PMC4607534; doi:10.1093/gbe/evv178)
Supplement: Supplementary Data [file supp_evv178_Suppl_Material_revised_version.docx]

Supplemental Materials and Methods, supplementary Tables S1−S5, and supplementary Figures S1−S9

**Insights into the evolutionary relationships of LytA autolysin and Ply pneumolysin-like genes in *Streptococcus pneumoniae* and related streptococci**

María Morales, Antonio J. Martín-Galiano, Mirian Domenech and Ernesto García*

**Materials and Methods**

**Bacterial strains, growth conditions and transformation**

Pneumococcal strains were grown in C medium ([Lacks and Hotchkiss 1960](#_ENREF_12)) either supplemented (C + Y) or not supplemented with 0.08% yeast extract. *Escherichia coli* strains were grown in Luria-Bertani medium ([Sambrook and Russell 2001](#_ENREF_19)). For *in vitro* biofilms assays, we used the non-encapsulated pneumococcal strain R6 and its derivatives: R924 (*lytA*::*kan*) ([Moscoso and Claverys 2004](#_ENREF_14)), P206 (*plyA*), and P259 (*plyA* *lytA*::*kan*). The following *E. coli* strains were used: DH5α (*supE44* Δ*lacU169* (φ80 *lacZ*ΔM15) *hsdR17 recA1 endA1 gyrA96 thi-1 relA1*) ([Hanahan 1983](#_ENREF_7)), and BL21 (DE3) (*hsdS gal* (λcI*ts857 ind1 S*am7 *nin5 lacUV5-*T7 gene *1*) (Novagen). Transformation of *E. coli* strains was performed by the rubidium chloride method ([Sambrook and Russell 2001](#_ENREF_19)).

**Biofilm formation assay and quantification**

The optimal conditions for biofilm formation by pneumococcal cells have been previously described ([Moscoso, et al. 2006](#_ENREF_16)). Cells were grown in C medium to an optical density at 550 nm (OD_550_) of 0.5–0.6, sedimented by centrifugation, resuspended in an equal volume of C medium, and diluted 1/10. Aliquots of 200 μl were dispensed into each well of polystyrene microtiter plates (Corning, New York, USA). After 6 h of incubation at 34°C, the biofilm formed was stained with 0.2% crystal violet and rinsed three times with distilled water to remove non-adherent bacteria. After solubilizing the biofilm in 95% ethanol, the OD_595_ was determined using an Anthos 2020 microplate absorbance reader (Anthos Labtec Instruments, Wals, Austria).

**PCR amplification and cloning of *lytA* alleles**

Routine DNA manipulations were performed essentially as described elsewhere ([Sambrook and Russell 2001](#_ENREF_19)). Restriction endonucleases and T4 DNA ligase were purchased from Takara Bio and used according to the recommendations of the suppliers. DNA fragments were purified using the Geneclean II kit (Bio 101). For PCR reactions, recombinant DNA polymerase from *Thermus thermophilus* HB27 was used (Biotools B&M Laboratories, Spain). For cloning, *lytA_Spn_* alleles coding for the NAM-amidase alleles 1, 2, 4, 5, or 7 (Supplemental Table S4) were PCR amplified using a mixture of Taq and Pfu DNA polymerases (Biotools B&M Laboratories, Spain) and oligonucleotides LytA-ATG and LytA-STOP ([Moscoso, et al. 2010](#_ENREF_15)). The products amplified by PCR were purified using a High Pure PCR product purification kit (Roche). The resultant DNA fragments (1,012 bp each) were digested with NdeI and BamHI and cloned into plasmid pT7-7 ([Tabor 1990](#_ENREF_21)). The ligation mixtures were used to transform *E. coli* DH5α. Next, the recombinant plasmids were introduced into *E. coli* BL21 (DE3). The accuracy of the different constructs was confirmed by completely sequencing the insert of the corresponding recombinant plasmid. All primers for PCR amplification and nucleotide sequencing were purchased from Sigma-Aldrich.

**Overproduction and purification of LytA alleles, and assay of cell wall lytic activity**

For overproduction of LytA NAM-amidases, *E. coli* BL21 (DE3) cells harboring the appropriate plasmid were incubated in Luria-Bertani medium with ampicillin (100 μg ml^−1^) up to an optical density at 600 nm of 0.4. At this time point, isopropyl-β-D-thiogalactopyranoside (0.1 mM) was added, and incubation continued for 4 h at 37°C with shaking. The cultures were centrifuged, and the bacteria were resuspended in 20 mM sodium phosphate buffer (pH 6.9) and disrupted by two 10 s periods of sonication. After centrifugation (10,000 × *g*, 10 min, 4°C), the supernatant was loaded onto a DEAE-cellulose column and NAM-amidases were purified as previously described ([Sánchez-Puelles, et al. 1992](#_ENREF_20)). LytA concentration was determined spectrophotometrically using a molar absorption coefficient at 280 nm of 113,750 M^−1^ cm^−1^ ([Usobiaga, et al. 1996](#_ENREF_24)).

Pneumococcal cell walls were radioactively labeled with [*methyl*-^3^H]choline and assays for cell wall lytic (NAM-amidase) activity were conducted according to standard procedures described elsewhere ([Mosser and Tomasz 1970](#_ENREF_17); [Romero, et al. 2004](#_ENREF_18)). One unit of amidase activity was defined as the amount of enzyme needed to catalyze the hydrolysis (solubilization) of 1 μg of cell wall material in 10 min at 37°C.

**Statistical analysis**

The data for NAM-amidase activity include the mean ± standard error of at least three independent experiments, each performed in triplicate. Statistical significance was examined using the Student *t* test. For multiples comparisons, one-way analyses of variance (ANOVA) were performed, followed by Dunnet’s *post hoc* test when the ANOVA rejected the null hypothesis. Differences were considered statistically significant when P <0.05. The SAS 9.3 statistical package (SAS Institute, Cary, NC) was used for all analyses.

**Table S1.** Complete and draft genomic sequences of SMG and other relevant streptococci^a^.

| Species | Complete genomes | Draft genomes | Reference*^b^* |
| --- | --- | --- | --- |
| SMG*^c^* |  |  |  |
| *S. australis* | 0 | 2 | ([Willcox, et al. 2001](#_ENREF_27)) |
| *S. cristatus* | 0 | 2 | ([Handley, et al. 1991](#_ENREF_8)) |
| *S. dentisani* | 0 | 2 | ([Camelo-Castillo, et al. 2014](#_ENREF_4)) |
| *S. gordonii* | 1 | 0 | ([Kilian, et al. 1989](#_ENREF_11)) |
| *S. infantis* | 0 | 6 | ([Kawamura, et al. 1998](#_ENREF_10)) |
| *S. lactarius* | 0 | 0 | ([Martín, et al. 2011](#_ENREF_13)) |
| *S. mitis* | 1 | 30 | ([Andrewes and Horder 1906](#_ENREF_1)) |
| *S. oligofermentans* | 1 | 0 | ([Tong, et al. 2003](#_ENREF_23)) |
| *S. oralis* | 1 | 12 | ([Bridge and Sneath 1982](#_ENREF_3)) |
| *S. parasanguinis* | 2 | 7 | ([Whiley, et al. 1990](#_ENREF_25)) |
| *S. peroris* | 0 | 1 | ([Kawamura, et al. 1998](#_ENREF_10)) |
| *S. pneumoniae* | 27 | 275 | ([Chester 1901](#_ENREF_5)) |
| *S. pseudopneumoniae* | 1 | 6 | ([Arbique, et al. 2004](#_ENREF_2)) |
| *S. rubneri* | 0 | 0 | ([Huch, et al. 2013](#_ENREF_9)) |
| *S. sanguinis* | 1 | 21 | ([White and Niven 1946](#_ENREF_26)) |
| *S. sinensis* | 0 | 1 | ([Woo, et al. 2002](#_ENREF_28)) |
| *S. tigurinus* | 0 | 4 | ([Zbinden, et al. 2012](#_ENREF_29)) |
| *S. troglodytidis* | 0 | 0 | ([Zhang, et al. 2013](#_ENREF_30)) |
| Other streptococci |  |  |  |
| *S. agalactiae* | 18 | 295 |  |
| *S. anginosus* | 4 | 10 |  |
| *S. mutans* | 4 | 161 |  |
| *S. pyogenes* | 30 | 209 |  |
| *S. suis* | 20 | 87 |  |
| *S. thermophilus* | 7 | 13 |  |
| Other species*^d^* | 37 | 167 |  |

**a**. Genomic sequences available at the National Center for Biotechnology Information (NCBI) (last date accessed, 25 October 2014).

**b.** Publication corresponds only to SMG.

**c.** *Streptococcus massiliensis* has also been proposed to belong to SMG but its phylogenetic position is still unclear ([Glazunova, et al. 2006](#_ENREF_6)).

**d.** *S. anginosus, S. caballi, S. canis, S. castoreus, S. constellatus, S. cricetus, D. devriesei, S. didelphis, S. downei, S. dysgalactiae, S. equi, S. equinus*, *S. ferus, S. gallolyticus, S. henryi, S. hyovaginalis, S. ictaluri, S. infantarius*, *S. iniae, S. intermedius, S. lutetiensis, S. macacae, S. macedonicus,S. marimammalium, S. massiliensis, S. merionis, S. minor, S. orisratti, S. ovis, S. parauberis, S. pasteurianus, S. plurextorum, S. porci, S. porcinus, S. pseudoporcinus, S. ratti, S. salivarius, S. thoraltensis, S. uberis, S. urinalis S. vestibularis,*and *Streptococcus* sp.

**Table S2.**

See separate file

**Table S3.**

See separate file

**Table S4.** Correspondence between *lytA* and LytA alleles of *S. pneumoniae*

| *lytA_Spn_* allele (Fam_)*^a^*  (Acc. No.) | LytA*_Spn_* Allele*^b^* | Specific activity | |
| --- | --- | --- | --- |
|  |  | (Units mg^−1^)*^c^* | Significant (*P* <0.05)*^d^* |
| 1 (B) (AE007317) | 1 | 1.3 × 10^6^ ± 0.97 × 10^5^ | allele 1 vs. 7: Yes |
| 2 (B) (AE005672) | 2 | 1.3 × 10^6^ ± 2.2 × 10^5^ | allele 2 vs. 7: Yes |
| 3 (A) (CP002176) | 3 |  |  |
| 4 (B) (FQ312030) | 4 | 1.2 × 10^6^ ± 1.5 × 10^5^ | allele 4 vs. 7: Yes |
| 5 (A) (AGPA01000015) | 5 | 1.6 × 10^6^ ± 1.9 × 10^5^ | allele 5 vs. 7: No |
| 6 (A) (AWTX01000004) | 5 |  |  |
| 7 (A) (FM211187) | 5 |  |  |
| 8 (A) (FQ312027) | 5 |  |  |
| 9 (A) (ALAZ01000017) | 6 |  |  |
| 10 (A) (FQ312045) | 2 |  |  |
| 11 (A) (CP002121) | 7 | 1.9 × 10^6^ ± 2.1 × 10^5^ |  |
| 12 (B) (AGID01000009) | 2 |  |  |
| 13 (A) (CP001845) | 2 |  |  |
| 14 (B) (AIKV01000007) | 2 |  |  |
| 15 (A) (CP000920) | 5 |  |  |
| 16 (B) (CP000918) | 2 |  |  |
| 17 (B) (ALCR01000016) | 2 |  |  |
| 18 (B) (AFAX01000007) | 8 |  |  |
| 19 (B) (ALCV01000008) | 5 |  |  |
| 20 (B) (ALBF01000021) | 2 |  |  |
| 21 (B) (AQTO01000004) | 9 |  |  |
| 22 (A) (AKQY01000003) | 10 |  |  |
| 23 (A) (ALBM01000008) | 11 |  |  |
| 24 (A) (AILM01000015) | 2 |  |  |
| 25 (A) (AILB01000008) | 12 |  |  |
| 26 (A) (AIKT01000015) | 5 |  |  |
| 27 (A) (ALBP01000011) | 13 |  |  |
| 28 (A) (ALBK01000014) | 6 |  |  |
| 29 (A) (ABAC01000008) | 7 |  |  |
| 30 (A) (ABAB01000008) | − |  |  |

*^a^* Fam_A and Fam_B of *lytA_Spn_* alleles have been described elsewhere ([Morales, et al. 2010](#_ENREF_1)).

*^b^* The *lytA*_30-_*_Spn_* allele was not translated into protein since it may contain a sequencing error.

*^c^* Expressed as the mean ± the standard error of the mean.

*^d^* For multiples comparisons, one-way analyses of variance (ANOVA) were performed, followed by Dunnet’s *post hoc* test when the ANOVA rejected the null hypothesis. Differences were considered statistically significant when P <0.05.

**References**

Morales M, et al. 2010. Evidence of localized prophage-host recombination in the lytA gene encoding the major pneumococcal autolysin. J Bacteriol 192:2624−2632.**Nucleotide sequences of the *lytA_Spn_* alleles analyzed in this study**

>1_SPN

ATGGAAATTAATGTGAGTAAATTAAGAACAGATTTGCCTCAAGTCGGCGTGCAACCATATAGGCAAGTACACGCACACTCAACTGGGAATCCGCATTCAACCGTACAGAATGAAGCGGATTATCACTGGCGGAAAGACCCAGAATTAGGTTTTTTCTCGCACATTGTTGGGAACGGTTGCATCATGCAGGTAGGACCTGTTGATAATGGTGCCTGGGACGTTGGGGGCGGTTGGAATGCTGAGACCTATGCAGCGGTTGAACTGATTGAAAGCCATTCAACCAAAGAAGAGTTCATGACGGACTACCGCCTTTATATCGAACTCTTACGCAATCTAGCAGATGAAGCAGGTTTGCCGAAAACGCTTGATACAGGGAGTTTAGCTGGAATTAAAACGCACGAGTATTGCACGAATAACCAACCAAACAACCACTCAGACCACGTTGACCCTTATCCATATCTTGCTAAATGGGGCATTAGCCGTGAGCAGTTTAAGCATGATATTGAGAACGGCTTGACGATTGAAACAGGCTGGCAGAAGAATGACACTGGCTACTGGTACGTACATTCAGACGGCTCTTATCCAAAAGACAAGTTTGAGAAAATCAATGGCACTTGGTACTACTTTGACAGTTCAGGCTATATGCTTGCAGACCGCTGGAGGAAGCACACAGACGGCAACTGGTACTGGTTCGACAACTCAGGCGAAATGGCTACAGGCTGGAAGAAAATCGCTGATAAGTGGTACTATTTCAACGAAGAAGGTGCCATGAAGACAGGCTGGGTCAAGTACAAGGACACTTGGTACTACTTAGACGCTAAAGAAGGCGCCATGGTATCAAATGCCTTTATCCAGTCAGCGGACGGAACAGGCTGGTACTACCTCAAACCAGACGGAACACTGGCAGACAGGCCAGAATTCACAGTAGAGCCAGATGGCTTGATTACAGTAAAATAA

>2_SPN

ATGGAAATTAATGTGAGTAAATTAAGAACAGATTTGCCTCAAGTCGGCGTGCAACCATATAGGCAAGTACACGCACACTCAACTGGGAATCCGCATTCAACCGTACAGAATGAAGCGGATTATCACTGGCGGAAAGACCCAGAATTAGGTTTTTTCTCGCACATTGTTGGGAACGGTTGCATCATGCAGGTAGGACCTGTTGATAATGGTGCCTGGGACGTTGGGGGCGGTTGGAATGCTGAGACCTATGCAGCGGTTGAACTGATTGAAAGCCATTCAACCAAAGAAGAGTTCATGACGGACTACCGCCTTTATATCGAACTCTTACGCAATCTAGCAGATGAAGCAGGTTTGCCGAAAACGCTTGATACAGGGAGTTTAGCTGGAATTAAAACGCACGAGTATTGCACGAATAACCAACCAAACAACCACTCAGACCACGTTGACCCTTATCCATATCTTGCTAAATGGGGCATTAGCCGTGAGCAGTTTAAGCATGATATTGAGAACGGCTTGACGATTGAAACAGGCTGGCAGAAGAATGACACTGGCTACTGGTACGTACATTCAGACGGCTCTTATCCAAAAGACAAGTTTGAGAAAATCAATGGCACTTGGTACTACTTTGACAGTTCAGGCTATATGCTTGCAGACCGCTGGAGGAAGCACACAGACGGCAACTGGTACTGGTTCGACAACTCAGGCGAAATGGCTACAGGCTGGAAGAAAATCGCTGATAAGTGGTACTATTTCAACGAAGAAGGTGCCATGAAGACAGGCTGGGTCAAGTACAAGGACACTTGGTACTACTTAGACGCTAAAGAAGGCGCCATGGTATCAAATGCCTTTATCCAGTCAGCGGACGGAACAGGCTGGTACTACCTCAAACCAGACGGAACACTGGCAGACAAGCCAGAATTCACAGTAGAGCCAGATGGCTTGATTACAGTAAAATAA

>3_SPN

ATGGAAATTAATGTGAGTAAATTAAGAACAGATTTGCCTCAAGTCGGCGTGCAACCATATAGGCAAGTACACGCACACTCAACTGGGAATCCGCATTCAACCGTACAGAATGAAGCGGATTATCACTGGCGGAAAGACCCAGAATTAGGTTTTTTCTCGCACATTGTTGGGAACGGTTGCATCATGCAGGTAGGACCTGTTGATAATGGTGCCTGGGACGTTGGGGGCGGTTGGAATGCTGAGACCTATGCAGCGGTTGAACTGATTGAAAGCCATTCAACTAAAGAAGAGTTCATGACGGACTACCGCCTTTATATCGAACTCTTACGCAATCTAGCAGATGAAGCAGGTTTGCCGAAAACGCTTGATACAGGGAGTTTAGCTGGAATTAAAACGCACGAGTATTGCACGAATAACCAACCAAACAACCACTCAGACCATGTGGATCCATACCCTTACTTGGCAAAATGGGGCATTAGCCGTGAGCAGTTTAAGTATGATATTGAGAACGGCTTGACGATTGAAACAGGCTGGCAGAAGAATGACACTGGCTACTGGTACGTACATTCAGACGGCTCTTATCCAAAAGACAAGTTTGAGAAAATCAATGGCACTTGGTACTACTTTGACAGTTCAGGCTATATGCTTGCAGACCGCTGGAGGAAGCACACAGACGGCAACTGGTACTGGTTCGACAACTCAGGCGAAATGGCTACAGGCTGGAAGAAAATCGCTGAGAAGTGGTACTATTTCAACGAAGAAGGTGCCATGAAGACAGGCTGGGTCAAGTACAAGGACACTTGGTACTACTTAGACGCTAAAGAAGGCGCCATGGTATCAAATGCCTTTATCCAGTCAGCGGACGGAACAGGCTGGTACTACCTCAAACCAGACGGAACACTGGCAGACAAGCCAGAATTCACAGTAGAGCCAGATGGCTTGATTACAGTTAAATAA

>4_SPN

ATGGAAATTAATGTGAGTAAATTAAGAACAGATTTGCCTCAAGTCGGCGTGCAACCATATAGGCAAGTACACGCACACTCAACTGGGAATCCGCATTCAACCGTACAGAATGAAGCGGATTATCACTGGCGGAAAGACCCAGAATTAGGTTTTTTCTCGCACATTGTTGGGAACGGTTGCATCATGCAGGTAGGACCTGTTGATAATGGTGCCTGGGACGTTGGGGGCGGTTGGAATGCTGAGACCTATGCAGCGGTTGAACTGATTGAAAGCCATTCAACCAAAGAAGAGTTCATGACGGACTACCGCCTTTATATCGAACTCTTACGCAATCTAGCAGATGAAGCAGGTTTGCCGAAAACGCTTGATACAGGGAGTTTAGCTGGAATTAAAACGCACGAGTATTGCACGAATAACCAACCAAACAACCACTCAGACCACGTTGACCCTTATCCATATCTTGCTAAATGGGGCATTAGCCGTGAGCAGTTTAAGCATGATATTGAGAACGGCTTGACGATTGAAACAGGCTGGCAGAAGAATGACACTGGCTACTGGTACGTACATTCAGACGGCTCTTATCCAAAAGACAAGTTTGAGAAAATCAATGGCACTTGGTACTACTTTGACAGTTCAGGCTATATGCTTGCAGACCGCTGGAGGAAGCACACAGACGGCAACTGGTACTGGTTCGACAACTCAGGCGAAATGGCTACAGGCTGGAAGAAAATCGCTGATAAGTGGTACTATTTCAACGAAGAAGGTGCCATGAAGACAGGCTGGGTCAAGTACAAGGACACTTGGTACTACTTAGACGCTAAAGAAGGCGCCATGGTATCAAATGCCTTTATCCAGTCAGCGGACGGAACAGGCTGGTACTACCTCAAACCAGACGGAACACTGGCAGACAAGCCAGACTTCACAGTAGAGCCTGAAGGCTTGATTACAGTAAAATAA

>5_SPN

atggaaattaatgtgagtaaattaagaacagatttgcctcaagtcggcgtgcaaccatataggcaagtacacgcacactcaactgggaatccgcattcaaccgtacagaatgaagcggattatcactggcggaaagacccagaattaggttttttctcgcacattgttgggaacggttgcatcatgcaggtaggacctgttgataatggtgcctgggacgttgggggcggttggaatgctgagacctatgcagcggttgaactgattgaaagccattcaactaaagaagagttcatgacggactaccgtctttatatcgaactcttacgcaatctagcagatgaagcaggtttgccgaaaacgcttgatacagggagtttagctggaattaaaacgcacgagtattgcacgaataaccaaccaaacaaccactcagaccatgtggatccatacccttacttggcaaaatggggcattagccgtgagcagtttaagtatgatattgagaacggcttgacgattgaaacaggctggcagaagaatgacactggctactggtacgtacattcagacggctcttatccaaaagacaagtttgagaaaatcaatggcacttggtactactttgacagttcaggctatatgcttgcagaccgctggaggaagcacacagacggcaactggtactggttcgacaactcaggtgaaatggctacaggctggaagaaaatcgctgataagtggtactatttcaacgaagaaggtgccatgaagacaggctgggtcaagtacaaggacacttggtactacttagacgctaaagaaggcgccatggtatcaaacgcctttatccagtcagcggacggaacaggctggtactacctcaaaccagacggaacactggcagacaagccagaattcacagtagagccagatggcttgattacagtaaaataa

>6_SPN

ATGGAAATTAATGTGAGTAAATTAAGAACAGATTTGCCTCAAGTCGGCGTGCAACCATATAGGCAAGTACACGCACACTCAACTGGGAATCCGCATTCAACCGTACAGAATGAAGCGGATTATCACTGGCGGAAAGACCCAGAATTAGGTTTTTTCTCGCACATTGTTGGGAACGGTTGCATCATGCAGGTAGGACCTGTTGATAATGGTGCCTGGGACGTTGGGGGCGGTTGGAATGCTGAGACCTATGCAGCGGTTGAACTGATTGAAAGCCATTCAACTAAAGAAGAGTTCATGACGGACTACCGCCTTTATATCGAACTCTTACGCAATCTAGCAGATGAAGCAGGTTTGCCGAAAACGCTTGATACAGGGAGTTTAGCTGGAATTAAAACGCACGAGTATTGCACGAATAACCAACCAAACAACCACTCAGACCATGTGGATCCATACCCTTACTTGGCAAAATGGGGCATTAGCCGTGAGCAGTTTAAGTATGATATTGAGAACGGCTTGACGATTGAAACAGGCTGGCAGAAGAATGACACTGGCTACTGGTACGTACATTCAGACGGCTCTTATCCAAAAGACAAGTTTGAGAAAATCAATGGCACTTGGTACTACTTTGACAGTTCAGGCTATATGCTTGCAGACCGCTGGAGGAAGCACACAGACGGCAACTGGTACTGGTTCGACAACTCAGGCGAAATGGCTACAGGCTGGAAGAAAATCGCTGATAAGTGGTACTATTTCAACGAAGAAGGTGCAATGAAGACAGGCTGGGTCAAGTACAAGGACACTTGGTACTACTTAGACGCTAAAGAAGGCGCCATGGTATCAAATGCCTTTATCCAGTCAGCGGACGGAACAGGCTGGTACTACCTCAAACCAGACGGAACACTGGCAGACAAGCCAGAATTCACAGTAGAGCCAGATGGCTTGATTACAGTAAAATAA

>7_SPN

ATGGAAATTAATGTGAGTAAATTAAGAACAGATTTGCCTCAAGTCGGCGTGCAACCATATAGGCAAGTACACGCACACTCAACTGGGAATCCGCATTCAACCGTACAGAATGAAGCGGATTATCACTGGCGGAAAGACCCAGAATTAGGTTTTTTCTCGCACATTGTTGGGAACGGTTGCATCATGCAGGTAGGACCTGTTGATAATGGTGCCTGGGACGTTGGGGGCGGTTGGAATGCTGAGACCTATGCAGCGGTTGAACTGATTGAAAGCCATTCAACTAAAGAAGAGTTCATGACGGACTACCGCCTTTATATCGAACTCTTACGCAATCTAGCAGATGAAGCAGGTTTGCCGAAAACGCTTGATACAGGGAGTTTAGCTGGAATTAAAACGCACGAGTATTGCACGAATAACCAACCAAACAACCACTCAGACCATGTGGATCCATACCCTTACTTGGCAAAATGGGGCATTAGCCGTGAGCAGTTTAAGTATGATATTGAGAACGGCTTGACGATTGAAACAGGCTGGCAGAAGAATGACACTGGCTACTGGTACGTACATTCAGACGGCTCTTATCCAAAAGACAAGTTTGAGAAAATCAATGGCACTTGGTACTACTTTGACAGTTCAGGCTATATGCTTGCAGACCGCTGGAGGAAGCACACAGACGGCAACTGGTACTGGTTCGACAACTCAGGCGAAATGGCTACAGGCTGGAAGAAAATCGCTGATAAGTGGTACTATTTCAACGAAGAAGGTGCCATGAAGACAGGCTGGGTCAAGTACAAGGACACTTGGTACTACTTAGACGCTAAAGAAGGCGCCATGGTATCAAATGCCTTTATCCAGTCAGCGGACGGAACAGGCTGGTACTACCTCAAACCAGACGGAACACTGGCAGACAAGCCAGAATTCACAGTAGAGCCAGATGGCTTGATTACAGTAAAATAA

>8_SPN

ATGGAAATTAATGTGAGTAAATTAAGAACAGATTTGCCTCAAGTCGGCGTGCAACCATATAGGCAAGTACACGCACACTCAACTGGGAATCCGCATTCAACCGTACAGAATGAAGCGGATTATCACTGGCGGAAAGACCCAGAATTAGGTTTTTTCTCGCACATTGTTGGGAACGGTTGCATCATGCAGGTAGGACCTGTTGATAATGGTGCCTGGGACGTTGGGGGCGGTTGGAATGCTGAGACCTATGCAGCGGTTGAACTGATTGAAAGCCATTCAACTAAAGAAGAGTTCATGACGGACTACCGCCTTTATATCGAACTCTTACGCAATCTAGCAGATGAAGCAGGTTTGCCGAAAACGCTTGATACAGGGAGTTTAGCTGGAATTAAAACGCACGAGTATTGCACGAATAACCAACCAAACAACCACTCAGACCATGTGGATCCATACCCTTACTTGGCAAAATGGGGCATTAGCCGTGAGCAGTTTAAGTATGATATTGAGAACGGCTTGACGATTGAAACAGGCTGGCAGAAGAATGACACTGGCTACTGGTACGTACATTCAGACGGCTCTTATCCAAAAGACAAGTTTGAGAAAATCAATGGCACTTGGTACTACTTTGACAGTTCAGGCTATATGCTTGCAGACCGCTGGAGGAAGCACACAGACGGCAACTGGTACTGGTTCGACAACTCAGGCGAAATGGCTACAGGCTGGAAGAAAATCGCTGATAAGTGGTACTATTTCAACGAAGAAGGTGCCATGAAGACAGGCTGGGTCAAGTACAAGGACACTTGGTACTACTTAGACGCTAAAGAAGGCGCCATGGTATCAAATGCTTTTATCCAGTCAGCGGACGGAACAGGCTGGTACTACCTCAAACCAGACGGAACACTGGCAGACAAGCCAGAATTCACAGTAGAGCCAGATGGCTTGATTACAGTAAAATAA

>9_SPN

ATGGAAATTAATGTGAGTAAATTAAGAACAGATTTGCCTCAAGTCGGCGTGCAACCATATAGGCAAGTACACGCACACTCAACTGGGAATCCGCATTCAACCGTACAGAATGAAGCGGATTATCACTGGCGGAAAGACCCAGAATTAGGTTTTTTCTCGCACATTGTTGGGAACGGTTGCATCATGCAGGTAGGACCTGTTGATAATGGTGCCTGGGACGTTGGGGGCGGTTGGAATGCTGAGACCTATGCAGCGGTTGAACTGATTGAAAGCCATTCAACTAAAGAAGAGTTCATGACGGACTACCGCCTTTATATCGAACTCTTACGCAATCTAGCAGATGAAGCAGGTTTGCCGAAAACGCTTGATACAGGGAGTTTAGCTGGAATTAAAACGCACGAGTATTGCACGAATAACCAACCAAACAACCACTCAGACCATGTGGATCCATACCCTTACTTGGCAAAATGGGGCATTAGCCGTGAGCAGTTTAAGCATGATATTGAGAACGGCTTGACGATTGAAACAGGCTGGCAGAAGAATGACACTGGCTACTGGTACGTACATTCAGACGGCTCTTATCCAAAAGACAAGTTTGAGAAAATCAATGGCACTTGGTACTACTTTGACAGTTCAGGCTATATGCTTGCAGACCGCTGGAGGAAGCACACAGACGGCAACTGGTACTGGTTCGACAACTCAGGCGAAATGGCTACAGGCTGGAAGAAAATCGCTGAGAAGTGGTACTATTTTGATGTAGAAGGTGCCATGAAGACAGGCTGGGTCAAGTACAAGGACACTTGGTACTACTTAGACGCTAAAGAAGGCGCCATGGTATCAAATGCCTTTATCCAGTCAGCGGACGGAACAGGCTGGTACTACCTCAAACCAGACGGAACACTGGCAGATAAACCAGAGTTCACAGTAGAGCCAGATGGCTTGATTACAGTAAAATAA

>10_SPN

ATGGAAATTAATGTGAGTAAATTAAGAACAGATTTGCCTCAAGTCGGCGTGCAACCATATAGGCAAGTACACGCACACTCAACTGGGAATCCGCATTCAACCGTACAGAATGAAGCGGATTATCACTGGCGGAAAGACCCAGAATTAGGTTTTTTCTCGCACATTGTTGGGAACGGTTGCATCATGCAGGTAGGACCTGTTGATAATGGTGCCTGGGACGTTGGGGGCGGTTGGAATGCTGAGACCTATGCAGCGGTTGAACTGATTGAAAGCCATTCAACTAAAGAAGAGTTCATGACGGACTACCGCCTTTATATCGAACTCTTACGCAATCTAGCAGATGAAGCAGGTTTGCCGAAAACGCTTGATACAGGGAGTTTAGCTGGAATTAAAACGCACGAGTATTGCACGAATAACCAACCAAACAACCACTCAGACCATGTGGATCCATACCCTTACTTGGCAAAATGGGGCATTAGCCGTGAGCAGTTTAAGCATGATATTGAGAACGGCTTGACGATTGAAACAGGCTGGCAGAAGAATGACACTGGCTACTGGTACGTACACTCAGACGGCTCTTATCCAAAAGACAAGTTTGAGAAAATCAATGGCACTTGGTACTACTTTGACAGTTCAGGCTATATGCTTGCAGACCGCTGGAGGAAGCACACAGACGGCAACTGGTACTGGTTCGACAACTCAGGCGAAATGGCTACAGGCTGGAAGAAAATCGCTGATAAGTGGTACTATTTCAACGAAGAAGGTGCCATGAAGACAGGCTGGGTCAAGTACAAGGACACTTGGTACTACTTAGACGCTAAAGAAGGCGCCATGGTATCAAATGCCTTTATCCAGTCAGCGGACGGAACAGGCTGGTACTACCTCAAACCAGACGGAACACTGGCAGACAAGCCAGAATTCACAGTAGAGCCAGATGGCTTGATTACAGTAAAATAA

>11_SPN

ATGGAAATTAATGTGAGTAAATTAAGAACAGATTTGCCTCAAGTCGGCGTGCAACCATATAGGCAAGTACACGCACACTCAACTGGGAATCCGCATTCAACCGTACAGAATGAAGCGGATTATCACTGGCGGAAAGACCCAGAATTAGGTTTTTTCTCGCACATTGTTGGGAACGGTTGCATCATGCAGGTAGGACCTGTTGATAATGGTGCCTGGGACGTTGGGGGCGGTTGGAATGCTGAGACCTATGCAGCGGTTGAACTGATTGAAAGCCATTCAACTAAAGAAGAGTTCATGACGGACTACCGCCTTTATATCGAACTCTTACGCAATCTAGCAGATGAAGCAGGTTTGCCGAAAACGCTTGATACAGGGAGTTTAGCTGGAATTAAAACGCACGAGTATTGCACGAATAACCAACCAAACAACCACTCAGACCATGTGGATCCATACCCTTACTTGGCAAAATGGGGCATTAGCCGTGAGCAGTTTAAGTATGATATTGAGAACGGCTTGACGATTGAAACAGGCTGGCAGAAGAATGACACTGGCTACTGGTACGTACATTCAGACGGCTCTTATCCAAAAGACAAGTTTGAGAAAATCAATGGCACTTGGTACTACTTTGACAGTTCAGGCTATATGCTTGCAGACCGCTGGAGGAAGCACACAGACGGCAACTGGTACTGGTTCGACAACTCAGGCGAAATGGCTACAGGCTGGAAGAAAATCGCTGAGAAGTGGTACTATTTTGATGTAGAAGGTGCCATGAAGACAGGCTGGGTCAAGTACAAGGACACTTGGTACTACTTAGACGCTAAAGAAGGCGCCATGGTATCAAATGCCTTTATCCAGTCAGCGGACGGAACAGGCTGGTACTACCTCAAACCAGACGGAACACTGGCAGACAAGCCAGAATTCACAGTAGAGCCAGATGGCTTGATTACAGTAAAATAA

>12_SPN

ATGGAAATTAATGTGAGTAAATTAAGAACAGATTTGCCTCAAGTCGGCGTGCAACCATATAGGCAAGTACACGCACACTCAACTGGGAATCCGCATTCAACCGTACAGAATGAAGCGGATTATCACTGGCGGAAAGACCCAGAATTAGGTTTTTTCTCGCACATTGTTGGGAACGGTTGCATCATGCAGGTAGGACCTGTTGATAATGGTGCCTGGGACGTTGGGGGCGGTTGGAATGCTGAGACCTATGCAGCGGTTGAACTTATTGAAAGCCATTCAACCAAAGAAGAGTTCATGACGGACTACCGCCTTTATATCGAACTCTTACGCAATCTAGCAGATGAAGCAGGTTTGCCGAAAACGCTTGATACAGGGAGTTTAGCTGGAATTAAAACGCACGAGTATTGCACGAATAACCAACCAAACAACCACTCAGACCACGTTGACCCTTATCCATATCTTGCTAAATGGGGCATTAGCCGTGAGCAGTTTAAGCATGATATTGAGAACGGCTTGACGATTGAAACAGGCTGGCAGAAGAATGACACTGGCTACTGGTACGTACATTCAGACGGCTCTTATCCAAAAGACAAGTTTGAGAAAATCAATGGCACTTGGTACTACTTTGACAGTTCAGGCTATATGCTTGCAGACCGCTGGAGGAAGCACACAGACGGCAACTGGTACTGGTTCGACAACTCAGGCGAAATGGCTACAGGCTGGAAGAAAATCGCTGATAAGTGGTACTATTTCAACGAAGAAGGTGCCATGAAGACAGGCTGGGTCAAGTACAAGGACACTTGGTACTACTTAGACGCTAAAGAAGGCGCCATGGTATCAAATGCCTTTATCCAGTCAGCGGACGGAACAGGCTGGTACTACCTCAAACCAGACGGAACACTGGCAGACAAGCCAGAATTCACAGTAGAGCCAGATGGCTTGATTACAGTAAAATAA

>13_SPN

ATGGAAATTAATGTGAGTAAATTAAGAACAGATTTGCCTCAAGTCGGCGTGCAACCATATAGGCAAGTACACGCACACTCAACTGGGAATCCGCATTCAACCGTACAGAATGAAGCGGATTATCACTGGCGGAAAGACCCAGAATTAGGTTTTTTCTCGCACATTGTTGGGAACGGTTGCATCATGCAGGTAGGACCTGTTGATAATGGTGCCTGGGACGTTGGGGGCGGTTGGAATGCTGAGACCTATGCAGCGGTTGAACTGATTGAAAGCCATTCAACTAAAGAAGAGTTCATGACGGACTACCGCCTTTATATCGAACTCTTACGCAATCTAGCAGATGAAGCAGGTTTGCCGAAAACGCTTGATACAGGGAGTTTAGCTGGAATTAAAACGCACGAGTATTGCACGAATAACCAACCAAACAACCACTCAGACCATGTGGATCCATACCCTTACTTGGCAAAATGGGGCATTAGCCGTGAGCAGTTTAAGCATGATATTGAGAACGGCTTGACGATTGAAACAGGCTGGCAGAAGAATGACACTGGCTACTGGTACGTACATTCAGACGGCTCTTATCCAAAAGACAAGTTTGAGAAAATCAATGGCACTTGGTACTACTTTGACAGTTCAGGCTATATGCTTGCAGACCGCTGGAGGAAGCACACAGACGGCAACTGGTACTGGTTCGACAACTCAGGCGAAATGGCTACAGGCTGGAAGAAAATCGCTGATAAGTGGTACTATTTCAACGAAGAAGGTGCCATGAAGACAGGCTGGGTCAAGTACAAGGACACTTGGTACTACTTAGACGCTAAAGAAGGCGCCATGGTATCAAATGCCTTTATCCAGTCAGCGGACGGAACAGGCTGGTACTACCTCAAACCAGACGGAACACTGGCAGACAAGCCAGAATTCACAGTAGAGCCAGATGGCTTGATTACAGTAAAATAA

>14_SPN

ATGGAAATTAATGTGAGTAAATTAAGAACAGATTTGCCTCAAGTCGGCGTGCAACCATATAGGCAAGTACACGCACACTCAACTGGGAATCCGCATTCAACCGTACAGAATGAAGCGGACTATCACTGGCGGAAAGACCCAGAATTAGGTTTTTTCTCGCACATTGTTGGGAACGGTTGCATCATGCAGGTAGGACCTGTTGATAATGGTGCCTGGGACGTTGGGGGCGGTTGGAATGCTGAGACCTATGCAGCGGTTGAACTGATTGAAAGCCATTCAACCAAAGAAGAGTTCATGACGGACTACCGCCTTTATATCGAACTCTTACGCAATCTAGCAGATGAAGCAGGTTTGCCGAAAACGCTTGATACAGGGAGTTTAGCTGGAATTAAAACGCACGAGTATTGCACGAATAACCAACCAAACAACCACTCAGACCACGTTGACCCTTATCCATATCTTGCTAAATGGGGCATTAGCCGTGAGCAGTTTAAGCATGATATTGAGAACGGCTTGACGATTGAAACAGGCTGGCAGAAGAATGACACTGGCTACTGGTACGTACATTCAGACGGCTCTTATCCAAAAGACAAGTTTGAGAAAATCAATGGCACTTGGTACTACTTTGACAGTTCAGGCTATATGCTTGCAGACCGCTGGAGGAAGCACACAGACGGCAACTGGTACTGGTTCGACAACTCAGGCGAAATGGCTACAGGCTGGAAGAAAATCGCTGATAAGTGGTACTATTTCAACGAAGAAGGTGCCATGAAGACAGGCTGGGTCAAGTACAAGGACACTTGGTACTACTTAGACGCTAAAGAAGGCGCCATGGTATCAAATGCCTTTATCCAGTCAGCGGACGGAACAGGCTGGTACTACCTCAAACCAGACGGAACACTGGCAGACAAGCCAGAATTCACAGTAGAGCCAGATGGCTTGATTACAGTAAAATAA

>15_SPN

ATGGAAATTAATGTGAGTAAATTAAGAACAGATTTGCCTCAAGTCGGCGTGCAACCATATAGGCAAGTACACGCACACTCAACTGGGAATCCGCATTCAACCGTACAGAATGAAGCGGATTATCACTGGCGGAAAGACCCAGAATTAGGTTTTTTCTCGCACATTGTTGGGAACGGTTGCATCATGCAGGTAGGACCTGTTGATAATGGTGCCTGGGACGTTGGGGGCGGTTGGAATGCTGAGACCTATGCAGCGGTTGAACTGATTGAAAGCCATTCAACTAAAGAAGAGTTCATGACGGATTACCGCCTTTATATCGAACTCTTACGCAATCTAGCAGATGAAGCAGGTTTGCCGAAAACGCTTGATACAGGGAGTTTAGCTGGAATTAAAACGCACGAGTATTGCACGAATAACCAACCAAACAACCACTCAGACCATGTGGATCCATACCCTTACTTGGCAAAATGGGGCATTAGCCGTGAGCAGTTTAAGTATGATATTGAGAACGGCTTGACGATTGAAACAGGCTGGCAGAAGAATGACACTGGCTACTGGTACGTACATTCAGACGGCTCTTATCCAAAAGACAAGTTTGAGAAAATCAATGGCACTTGGTACTACTTTGACAGTTCAGGCTATATGCTTGCAGACCGCTGGAGGAAGCACACAGACGGCAACTGGTACTGGTTCGACAACTCAGGCGAAATGGCTACAGGCTGGAAGAAAATCGCTGATAAGTGGTACTATTTCAACGAAGAAGGTGCCATGAAGACAGGCTGGGTCAAGTACAAGGACACTTGGTACTACTTAGACGCTAAAGAAGGCGCCATGGTATCAAATGCCTTTATCCAGTCAGCGGACGGAACAGGCTGGTACTACCTCAAACCAGACGGAACACTGGCAGACAAGCCAGAATTCACAGTAGAGCCAGATGGCTTGATTACAGTAAAATAA

>16_SPN

ATGGAAATTAATGTGAGTAAATTAAGAACAGATTTGCCTCAAGTCGGCGTGCAACCATATAGGCAAGTACACGCACACTCAACTGGGAATCCGCATTCAACCGTACAGAATGAAGCGGATTATCACTGGCGGAAAGACCCAGAATTAGGTTTTTTCTCGCACATTGTTGGGAACGGTTGCATCATGCAGGTAGGACCTGTTGATAATGGTGCCTGGGACGTTGGGGGCGGTTGGAATGCTGAGACCTATGCAGCGGTTGAACTGATTGAAAGCCATTCAACTAAAGAAGAGTTCATGACGGACTACCGCCTTTATATCGAACTCTTACGCAATCTAGCAGATGAAGCAGGTTTGCCGAAAACGCTTGATACAGGGAGTTTAGCTGGAATTAAAACGCACGAGTATTGCACGAATAACCAACCAAACAACCACTCAGACCACGTTGACCCTTATCCATATCTTGCTAAATGGGGCATTAGCCGTGAGCAGTTTAAGCATGATATTGAGAACGGCTTGACGATTGAAACAGGCTGGCAGAAGAATGACACTGGCTACTGGTACGTACATTCAGACGGCTCTTATCCAAAAGACAAGTTTGAGAAAATCAATGGCACTTGGTACTACTTTGACAGTTCAGGCTATATGCTTGCAGACCGCTGGAGGAAGCACACAGACGGCAACTGGTACTGGTTCGACAACTCAGGCGAAATGGCTACAGGCTGGAAGAAAATTGCTGATAAGTGGTACTATTTCAACGAAGAAGGTGCCATGAAGACAGGCTGGGTCAAGTACAAGGACACTTGGTACTACTTAGACGCTAAAGAAGGCGCCATGGTATCAAATGCCTTTATCCAGTCAGCGGACGGAACAGGCTGGTACTACCTCAAACCAGACGGAACACTGGCAGACAAGCCAGAATTCACAGTAGAGCCAGATGGCTTGATTACAGTAAAATAA

>17_SPN

ATGGAAATTAATGTGAGTAAATTAAGAACAGATTTGCCTCAAGTCGGCGTGCAACCATATAGGCAAGTACACGCACACTCAACTGGGAATCCGCATTCAACCGTACAGAATGAAGCGGATTATCACTGGCGGAAAGACCCAGAATTAGGTTTTTTCTCGCACATTGTTGGGAACGGTTGCATCATGCAGGTAGGACCTGTTGATAATGGTGCCTGGGACGTTGGGGGCGGTTGGAATGCTGAGACCTATGCAGCGGTTGAACTGATTGAAAGCCATTCAACCAAAGAAGAGTTCATGACGGACTACCGCCTTTATATCGAACTCTTACGCAATCTAGCAGATGAAGCAGGTTTGCCGAAAACGCTTGATACAGGGAGTTTAGCTGGAATTAAAACGCACGAGTATTGCACGAATAACCAACCAAACAACCACTCAGACCACGTTGACCCTTATCCATATCTTGCTAAATGGGGCATTAGCCGTGAGCAGTTTAAGCATGATATTGAGAACGGCTTGACGATTGAAACAGGCTGGCAGAAGAATGACACTGGCTACTGGTACGTACATTCAGACGGCTCTTATCCAAAAGACAAGTTTGAGAAAATCAATGGCACTTGGTACTACTTTGACAGTTCAGGCTATATGCTTGCAGACCGCTGGAGGAAGCACACAGACGGCAACTGGTACTGGTTCGACAACTCAGGCGAAATGGCTACAGGCTGGAAGAAAATTGCTGATAAGTGGTACTATTTCAACGAAGAAGGTGCCATGAAGACAGGCTGGGTCAAGTACAAGGACACTTGGTACTACTTAGACGCTAAAGAAGGCGCCATGGTATCAAATGCCTTTATCCAGTCAGCGGACGGAACAGGCTGGTACTACCTCAAACCAGACGGAACACTGGCAGACAAGCCAGAATTCACAGTAGAGCCAGATGGCTTGATTACAGTAAAATAA

>18_SPN

ATGGAAATTAATGTGAGTAAATTAAGAACAGATTTGCCTCAAGTCGGCGTGCAACCATATAGGCAAGTACACGCACACTCAACTGGGAATTCGCATTCAACCGTACAGAATGAAGCGGACTATCACTGGCGGAAAGACCCAGAATTAGGTTTTTTCTCGCACATTGTTGGGAACGGTTGCATCATGCAGGTAGGACCTGTTGATAATGGTGCCTGGGACGTTGGGGGCGGTTGGAATGCTGAGACCTATGCAGCGGTTGAACTGATTGAAAGCCATTCAACCAAAGAAGAGTTCATGACGGACTACCGCCTTTATATCGAACTCTTACGCAATCTAGCAGATGAAGCAGGTTTGCCGAAAACGCTTGATACAGGGAGTTTAGCTGGAATTAAAACGCACGAGTATTGCACGAATAACCAACCAAACAACCACTCAGACCACGTTGACCCTTATCCATATCTTGCTAAATGGGGCATTAGCCGTGAGCAGTTTAAGCATGATATTGAGAACGGCTTGACGATTGAAACAGGCTGGCAGAAGAATGACACTGGCTACTGGTACGTACATTCAGACGGCTCTTATCCAAAAGACAAGTTTGAGAAAATCAATGGCACTTGGTACTACTTTGACAGTTCAGGCTATATGCTTGCAGACCGCTGGAGGAAGCACACAGACGGCAACTGGTACTGGTTCGACAACTCAGGCGAAATGGCTACAGGCTGGAAGAAAATCGCTGATAAGTGGTACTATTTCAACGAAGAAGGTGCCATGAAGACAGGCTGGGTCAAGTACAAGGACACTTGGTACTACTTAGACGCTAAAGAAGGCGCCATGGTATCAAATGCCTTTATCCAGTCAGCGGACGGAACAGGCTGGTACTACCTCAAACCAGACGGAACACTGGCAGACAAGCCAGAATTCACAGTAGAGCCAGATGGCTTGATTACAGTAAAATAA

>19_SPN

ATGGAAATTAATGTGAGTAAATTAAGAACAGATTTGCCTCAAGTCGGCGTGCAACCATATAGGCAAGTACACGCACACTCAACTGGGAATCCGCATTCAACCGTACAGAATGAAGCGGATTATCACTGGCGGAAAGACCCAGAATTAGGTTTTTTCTCGCACATTGTTGGGAACGGTTGCATCATGCAGGTAGGACCTGTTGATAATGGTGCCTGGGACGTTGGGGGCGGTTGGAATGCTGAGACCTATGCAGCGGTTGAACTGATTGAAAGCCATTCAACCAAAGAAGAGTTCATGACGGACTACCGCCTTTATATCGAACTCTTACGCAATCTAGCAGATGAAGCAGGTTTGCCGAAAACGCTTGATACAGGGAGTTTAGCTGGAATTAAAACGCACGAGTATTGCACGAATAACCAACCAAACAACCACTCAGACCACGTTGACCCTTATCCATATCTTGCTAAATGGGGCATTAGCCGTGAGCAGTTTAAGTATGATATTGAGAACGGCTTGACGATTGAAACAGGCTGGCAGAAGAATGACACTGGCTACTGGTACGTACATTCAGACGGCTCTTATCCAAAAGACAAGTTTGAGAAAATCAATGGCACTTGGTATTACTTTGACAGTTCAGGCTATATGCTTGCAGACCGCTGGAGGAAGCACACAGACGGCAACTGGTACTGGTTCGACAACTCAGGCGAAATGGCTACAGGCTGGAAGAAAATCGCTGATAAGTGGTACTATTTCAACGAAGAAGGTGCCATGAAGACAGGCTGGGTCAAGTACAAGGACACTTGGTACTACTTAGACGCTAAAGAAGGCGCCATGGTATCAAATGCCTTTATCCAGTCAGCGGACGGAACAGGCTGGTACTACCTCAAACCAGACGGAACACTGGCAGACAAGCCAGAATTCACAGTAGAGCCAGATGGCTTGATTACAGTAAAATAA

>20_SPN

atggaaattaatgtgagtaaattaagaacagatttgcctcaagtcggcgtgcaaccatataggcaagtacacgcacactcaactgggaatccgcattcaaccgtacagaatgaagcggattatcactggcggaaagacccagaattaggttttttctcgcacattgttgggaacggttgcatcatgcaggtaggacctgttgataatggtgcctgggacgttggGggcggttggaatgctgagacctatgcagcggttgaactgattgaaagccattcaaccaaagaagagttcatgacggactaccgcctttatatcgaactcttacgcaatctagcagatgaagcaggtttgccgaaaacgcttgatacagggagtttagctggaattaaaacgcacgagtattgcacgaataaccaaccaaacaaccactcagaccacgttgacccttatccatatcttgctaaatggggcattagccgtgagcagtttaagcatgatattgagaacggcttgacgattgaaacaggctggcagaagaatgacactggctactggtacgtacattcagacggctcttatccaaaagacaagtttgagaaaatcaatggcacttggtactactttgacagttcaggctatatgcttgcagaccgctggaggaagcacacagacggcaattggtactggttcgacaactcaggcgaaatggctacaggctggaagaaaatcgctgataagtggtactatttcaacgaagaaggtgccatgaagacaggctgggtcaagtacaaggacacttggtactacttagacgctaaagaaggcgccatggtatcaaatgcctttatccagtcagcggacggaacaggctggtactacctcaaaccagacggaacactggcagacaagccagaattcacagtagagccagatggcttgattacagtaaaataa

>21_SPN

atggaaattaatgtgagtaaattaagaacagatttgcctcaagtcggcgtgcaaccatataggcaagtacacgcacactcaactgggaatccgcattcaaccgtacagaatgaagcggactatcactggcggaaagacccagaattaggttttttctcgcacattgttgggaacggttgcatcatgcaggtaggacctgttgataatggtgcctgggacgttgggggcggttggaatgctgagacctatgcagcggttgaactgattgaaagccattcaaccaaagaagagttcatgacggactaccgcctttatatcgaactcttacgcaatctagcagatgaagcaggtttgccgaaaacgcttgatacagggagtttagctggaattaaaacgcacgagtattgcacgaataaccaaccaaacaaccactcagaccacgttgacccttatccatatcttgctaaatggggcattagccgtgagcagtttaagcatgatattgagaacggcttgacgattgaaacaggctggcagaagaatgacactggctactggtacgtacattcagacggctcttatccaaaagacaagtttgagaaaatcaatggcacttggtactactttgacagttcaggctatatgcttgcagaccgctggaggaagcacacagacggcaactggtactggttcgacaactcaggcgaaatggctacaggctggaagaaaatcgctgataagtggtactatttcaacgaagaaggtgccatgaagacaggctgggtcaagtacaaggacacttggtactacttagacgctaaagaaggcgccatggtatcaaatgcctttatccagtcagcggacggaacaggctggtactacctcaaaccagacggaacactggcagacaagccagacttcacagtagagccagatggcttgattacagtaaaataa

>22_SPN

atggaaattaatgtgagtaaattaagaacagatttgcctcaagtcggcgtgcaaccatataggcaagtacacgcacactcaactgggaatccgcattcaaccgtacagaatgaagcggattatcactggcggaaagacccagaattaggttttttctcgcacattgttgggaacggttgcatcatgcaggtaggacctgttgataatggtgcctgggacgttgggggcggttggaatgctgagagttatgcagcggttgaactgattgaaagccattcaactaaagaagagttcatgacggactaccgcctttatatcgaactcttacgcaatctagcagatgaagcaggtttgccgaaaacgcttgatacagggagtttagctggaattaaaacgcacgagtattgcacgaataaccaaccaaacaaccactcagaccatgtggatccatacccttacttggcaaaatggggcattagccgtgagcagtttaagcatgatattgagaacggcttgacgattgaaacaggctggcagaagaatgacactggctactggtacgtacattcagacggctcttatccaaaagacaagtttgagaaaatcaatggcacttggtactactttgacagttcaggctatatgcttgcagaccgctggaggaagcacacagacggcaactggtactggttcgacaactcaggcgaaatggctacaggctggaagaaaatcgctgataagtggtactatttcaacgaagaaggtgccatgaagacaggctgggtcaagtacaaggacacttggtactacttagacgctaaagaaggcgccatggtatcaaatgcctttatccagtcagcggacggaacaggctggtactacctcaaaccagacggaacactggcagacaagccagaattcacagtagagccagatggcttgattacagtaaaataa

>23_SPN

atggaaattaatgtgagtaaattaagaacagatttgcctcaagtcggcgtgcaaccatataggcaagtacacgcacactcaactgggaatccgcattcaaccgtacagaatgaagcggattatcactggcggaaagacccagaattaggttttttctcgcacattgttgggaacggttgcatcatgcaggtaggacctgttgataatggtgcctgggacgttgggggcggttggaatgctgagacctatgcagcggttgaactgattgaaagccattcaactaaagaagagttcatgacggactaccgcctttatatcgaactcttacgcaatctagcagatgaagcaggtttgccgaaaacgcttgatacagggagtttagctggaattaaaacgcacgagtattgcacgaataaccaaccaaacaaccactcagaccatgtggatccatacccttacttggcaaaatggggcattagccgtgagcagtttaagtatgatattgagaacgacttgacgattgaaacaggctggcagaagaatgacactggctactggtacgtacattcagacggctcttatccaaaagacaagtttgagaaaatcaatggcacttggtactactttgacagttcaggctatatgcttgcagaccgctggaggaagcacacagacggcaactggtactggttcgacaactcaggcgaaatggctacaggctggaagaaaatcgctgataagtggtactatttcaacgaagaaggtgccatgaagacaggctgggtcaagtacaaggacacttggtactacttagacgctaaagaaggcgccatggtatcaaatgcctttatccagtcagcggacggaacaggctggtactacctcaaaccagacggaacactggcagacaagccagaattcacagtagagccagatggcttgattacagtaaaataa

>24_SPN

atggaaattaatgtgagtaaattaagaacagatttgcctcaagtcggcgtgcaaccatataggcaagtacacgcacactcaactgggaatccgcattcaaccgtacagaatgaagcggattatcactggcggaaagacccagaattaggttttttctcgcacattgtggggaacggttgcatcatgcaggtaggacctgttgataatggtgcctgggacgttgggggcggttggaatgctgagacctatgcagcggttgaactgattgaaagccattcaactaaagaagagttcatgacggactaccgcctttatatcgaactcttacgcaatctagcagatgaagcaggtttgccgaaaacgcttgatacagggagtttagctggaattaaaacgcacgagtattgcacgaataaccaaccaaacaaccactcagaccatgtggatccatacccttacttggcaaaatggggcattagccgtgagcagtttaagcatgatattgagaacggcttgacgattgaaacaggctggcagaagaatgacactggctactggtacgtacactcagacggctcttatccaaaagacaagtttgagaaaatcaatggcacttggtactactttgacagttcaggctatatgcttgcagaccgctggaggaagcacacagacggcaactggtactggttcgacaactcaggcgaaatggctacaggctggaagaaaatcgctgataagtggtactatttcaacgaagaaggtgccatgaagacaggctgggtcaagtacaaggacacttggtactacttagacgctaaagaaggcgccatggtatcaaatgcctttatccagtcagcggacggaacaggctggtactacctcaaaccagacggaacactggcagacaagccagaattcacagtagagccagatggcttgattacagtaaaataa

>25_SPN

atggaaattaatgtgagtaaattaagaacagatttgcctcaagtcggcgtgcaaccatataggcaagtacacgcacactcaactgggaatccgcattcaaccgtacagaatgaagcggattatcactgtcggaaagacccagaattaggttttttctcgcacattgttgggaacggttgcatcatgcaggtaggacctgttgataatggtgcctgggacgttgggggcggttggaatgctgagacctatgcagcggttgaactgattgaaagccattcaactaaagaagagttcatgacggactaccgcctttatatcgaactcttacgcaatctagcagatgaagcaggtttgccgaaaacgcttgatacagggagtttagctggaattaaaacgcacgagtattgcacgaataaccaaccaaacaaccactcagaccatgtggatccatacccttacttggcaaaatggggcattagccgtgagcagtttaagcatgatattgagaacggcttgacgattgaaacaggctggcagaagaatgacactggctactggtacgtacactcagacggctcttatccaaaagacaagtttgagaaaatcaatggcacttggtactactttgacagttcaggctatatgcttgcagaccgctggaggaagcacacagacggcaactggtactggttcgacaactcaggcgaaatggctacaggctggaagaaaatcgctgataagtggtactatttcaacgaagaaggtgccatgaagacaggctgggtcaagtacaaggacacttggtactacttagacgctaaagaaggcgccatggtatcaaatgcctttatccagtcagcggacggaacaggctggtactacctcaaaccagacggaacactggcagacaagccagaattcacagtagagccagatggcttgattacagtaaaataa

>26_SPN

atggaaattaatgtgagtaaattaagaacagatttgcctcaagtcggcgtgcaaccatataggcaagtacacgcacactcaactgggaatccgcattcaaccgtacagaatgaagcggattatcactggcggaaagacccagaattaggttttttctcgcacattgttgggaacggttgcatcatgcaggtaggacctgttgataatggtgcctgggacgttgggggcggttggaatgctgagacctatgcagcggttgaactgattgaaagccattcaactaaagaagagttcatgacggactaccgcctttatatcgaactcttacgcaatctagcagatgaagcaggtttgccgaaaacgcttgatacagggagtttagctggaattaaaacgcacgagtattgcacgaataaccaaccaaacaaccactcagaccatgtggatccatacccttacttggcaaaatggggcattagccgtgagcagtttaagtatgatattgagaacggcttgacgattgaaacaggctggcagaagaatgacactggctactggtacgtacattcagacggctcttatccaaaagacaagtttgagaaaatcaatggcacttggtactactttgacagttcaggctatatgcttgcagaccgctggaggaagcacacagacggcaactggtactggttcgacaactcaggtgaaatggctacaggctggaagaaaatcgctgataagtggtactatttcaacgaagaaggtgccatgaagacaggctgggtcaagtacaaggacacttggtactacttagacgctaaagaaggcgccatggtatcaaacgcctttatccagtcagcggacggaacaggctggtactacctcaaaccagacggaacactggcagacaagccagaattcacagtagagccagatggcttgattacagtaaaataa

>27_SPN

atggaaattaatgtgagtaaattaagaacagatttgcctcaagtcggcgtgcaaccatataggcaagtacacgcacactcaactgggaatccgcattcaaccgtacagaatgaagcggattatcactggcggaaagacccagaattaggttttttctcgcacattgttgggaacggttgcatcatgcaggtaggacctgttgataatggtgcctgggacgttgggggcggttggaatgctgagacctatgcagcggttgaactgattgaaagccattcaactaaagaagagttcatgacggactaccgcctttatatcgaactcttacgcaatctagcagatgaagcaggtttgccgaaaacgcttgatacagggagtttagctggaattaaaacgcacgagtattgcacgaataaccaaccaaacaaccactcagaccatgtggatccatacccttacttggcaaaatggggcattagccgtgagcagtttaagtatgatattgagaacggcttgacgattgaaacaggctggcagaagaatgacactggctactggtacgtacattcagacggctcttatccaaaagacaagtttgagaaaatcaatggcacttggtactactttgacagttcaggctatatgcttgcagaccgctggaggaagcacacagacggcaactggtactggttcgacaactcaggcgaaatggctacaggctggaagaaaatcgctgataagtggtactattttgatgtagaaggtgccatgaagacaggctgggtcaagtacaaggacacttggtactacttagacgctaaagaaggcgccatggtatcaaatgcctttatccagtcagcggacggaacaggctggtactacctcaaaccagacggaacactggcagataaaccagagttcacagtagagccagatggcttgattacagtaaaataa

>28_SPN

ATGGAAATTAATGTGAGTAAATTAAGAACAGATTTGCCTCAAGTCGGCGTGCAACCATATAGGCAAGTACACGCACACTCAACTGGGAATCCGCATTCAACCGTACAGAATGAAGCGGATTATCACTGGCGGAAAGACCCAGAATTAGGTTTTTTCTCGCACATTGTTGGGAACGGTTGCATCATGCAGGTAGGACCTGTTGATAATGGTGCCTGGGACGTTGGGGGCGGTTGGAATGCTGAGACCTATGCAGCGGTTGAACTGATTGAAAGCCATTCAACTAAAGAAGAGTTCATGACGGACTACCGCCTTTATATCGAACTCTTACGCAATCTAGCAGATGAAGCAGGTTTGCCGAAAACGCTTGATACAGGGAGCTTAGCTGGAATTAAAACGCACGAGTATTGCACGAATAACCAACCAAACAACCACTCAGACCATGTGGATCCATACCCTTACTTGGCAAAATGGGGCATTAGCCGTGAGCAGTTTAAGCATGATATTGAGAACGGCTTGACGATTGAAACAGGCTGGCAGAAGAATGACACTGGCTACTGGTACGTACATTCAGACGGCTCTTATCCAAAAGACAAGTTTGAGAAAATCAATGGCACTTGGTACTACTTTGACAGTTCAGGCTATATGCTTGCAGACCGCTGGAGGAAGCACACAGACGGCAACTGGTACTGGTTCGACAACTCAGGCGAAATGGCTACAGGCTGGAAGAAAATCGCTGAGAAGTGGTACTATTTTGATGTAGAAGGTGCCATGAAGACAGGCTGGGTCAAGTACAAGGACACTTGGTACTACTTAGACGCTAAAGAAGGCGCCATGGTATCAAATGCCTTTATCCAGTCAGCGGACGGAACAGGCTGGTACTACCTCAAACCAGACGGAACACTGGCAGATAAACCAGAGTTCACAGTAGAGCCAGATGGCTTGATTACAGTAAAATAA

>29_SPN

ATGGAAATTAATGTGAGTAAATTAAGAACAGATTTGCCTCAAGTCGGCGTGCAACCATATAGGCAAGTACACGCACACTCAACTGGGAATCCGCATTCAACCGTACAGAATGAAGCGGATTATCACTGGCGGAAAGACCCAGAATTAGGTTTTTTCTCGCACATTGTTGGGAACGGTTGCATCATGCAGGTAGGACCTGTTGATAATGGTGCCTGGGACGTTGGGGGCGGTTGGAATGCTGAGACCTATGCAGCGGTTGAACTGATTGAAAGCCATTCAACTAAAGAAGAGTTCATGACGGACTACCGCCTTTATATCGAACTCTTACGCAATCTAGCAGATGAAGCAGGTTTGCCGAAAACGCTTGATACAGGGAGTTTAGCTGGAATTAAAACGCACGAGTATTGCACGAATAACCAACCAAACAACCACTCAGACCATGTGGATCCATACCCTTACTTGGCAAAATGGGGCATTAGCCGTGAGCAGTTTAAGTATGATATTGAGAACGGCTTGACGATTGAAACAGGCTGGCAGAAGAATGACACTGGCTACTGGTACGTACATTCAGACGGCTCTTATCCAAAAGACAAGTTTGAGAAAATCAATGGCACTTGGTACTACTTTGACAGTTCAGGCTATATGCTTGCAGACCGCTGGAGGAAGCACACAGACGGCAACTGGTACTGGTTCGACAACTCAGGCGAAATGGCTACAGGCTGGAAGAAAATCGCTGAGAAGTGGTACTATTTTGATGTAGAAGGTGCCATGAAGACAGGCTGGGTCAAGTACAAGGACACTTGGTACTACTTAGACGCTAAAGAAGGCGCCATGGTATCAAATGCCTTTATCCAGTCAGCGGACGGAACAGGCTGGTACTACCTCAAACCAGACGGAACACTGGCAGACAAGCCAGAGTTCACAGTAGAGCCAGATGGCTTGATTACAGTAAAATAA

>30_SPN

ATGGAAATTAATGTGAGTAAATTAAGAACAGATTTGCCTCAAGTCGGCGTGCAACCATATAGGCAAGTACACGCACACTCAACTGGGAATCCGCATTCAACCGTACAGAATGAAGCGGATTATCACTGGCGGAAAGACCCAGAATTAGGTTTTTTCTCGCACATTGTTGGGAACGGTTGCATCATGCAGGTAGGACCTGTTGATAATGGTGCCTGGGACGTTGGGGGCGGTTGGAATGCTGAGACCTATGCAGCGGTTGAACTGATTGAAAGCCATTCAACTAAAGAAGAGTTCATGACGGACTACCGCCTTTATATCGAACTCTTACGCAATCTAGCAGATGAAGCAGGTTTGCCGAAAACGCTTGATACAGGGAGTTTAGCTGGAATTAAAACGCACGAGTATTGCACGAATAACCAACCAAACAACCACTCAGACCATGTGGATCCATACCCTTACTTGGCAAAATGGGGCATTAGCCGTGAGCAGTTTAAGCATGATATTGAGAACGGCTTGACGATTGAAACAGGCTGGCAGAAGAATGACACTGGCTACTGGTACGTACACTCAGACGGCTCTTATCCAAAAGACAAGTTTGAGAAAATCAATGGCACTTGGTACTACTTTGACAGTTCAGGCTATATGCTTGCAGACCGCTGGAGGAAGCACACAGACGGCAACTGGTACTGGTTCGACAACTCAGGCGAAATGGCTACAGGCTGGAAGAAAATCGCTGATAAGTGGTACTATTTCAACGAAGAAGGTGCCATGAAGACTTGAAGGGTCAAGTACAAGGACACTTGGTACTACTTAGACGCTAAAGAAGGCGCCATGGTATCAAATGCCTTTATCCAGTCAGCGGACGGAACAGGCTGGTACTACCTCAAACCAGACGGAACACTGGCAGACAAGCCAGAATTCACAGTAGAGCCAGATGGCTTGATTACAGTAAAATAA

**Amino acid sequences of the LytA*_Spn_* alleles analyzed in this study**

>1_SPN

MEINVSKLRTDLPQVGVQPYRQVHAHSTGNPHSTVQNEADYHWRKDPELGFFSHIVGNGCIMQVGPVDNGAWDVGGGWNAETYAAVELIESHSTKEEFMTDYRLYIELLRNLADEAGLPKTLDTGSLAGIKTHEYCTNNQPNNHSDHVDPYPYLAKWGISREQFKHDIENGLTIETGWQKNDTGYWYVHSDGSYPKDKFEKINGTWYYFDSSGYMLADRWRKHTDGNWYWFDNSGEMATGWKKIADKWYYFNEEGAMKTGWVKYKDTWYYLDAKEGAMVSNAFIQSADGTGWYYLKPDGTLADRPEFTVEPDGLITVK

>2_SPN

MEINVSKLRTDLPQVGVQPYRQVHAHSTGNPHSTVQNEADYHWRKDPELGFFSHIVGNGCIMQVGPVDNGAWDVGGGWNAETYAAVELIESHSTKEEFMTDYRLYIELLRNLADEAGLPKTLDTGSLAGIKTHEYCTNNQPNNHSDHVDPYPYLAKWGISREQFKHDIENGLTIETGWQKNDTGYWYVHSDGSYPKDKFEKINGTWYYFDSSGYMLADRWRKHTDGNWYWFDNSGEMATGWKKIADKWYYFNEEGAMKTGWVKYKDTWYYLDAKEGAMVSNAFIQSADGTGWYYLKPDGTLADKPEFTVEPDGLITVK

>3_SPN

MEINVSKLRTDLPQVGVQPYRQVHAHSTGNPHSTVQNEADYHWRKDPELGFFSHIVGNGCIMQVGPVDNGAWDVGGGWNAETYAAVELIESHSTKEEFMTDYRLYIELLRNLADEAGLPKTLDTGSLAGIKTHEYCTNNQPNNHSDHVDPYPYLAKWGISREQFKYDIENGLTIETGWQKNDTGYWYVHSDGSYPKDKFEKINGTWYYFDSSGYMLADRWRKHTDGNWYWFDNSGEMATGWKKIAEKWYYFNEEGAMKTGWVKYKDTWYYLDAKEGAMVSNAFIQSADGTGWYYLKPDGTLADKPEFTVEPDGLITVK

>4_SPN

MEINVSKLRTDLPQVGVQPYRQVHAHSTGNPHSTVQNEADYHWRKDPELGFFSHIVGNGCIMQVGPVDNGAWDVGGGWNAETYAAVELIESHSTKEEFMTDYRLYIELLRNLADEAGLPKTLDTGSLAGIKTHEYCTNNQPNNHSDHVDPYPYLAKWGISREQFKHDIENGLTIETGWQKNDTGYWYVHSDGSYPKDKFEKINGTWYYFDSSGYMLADRWRKHTDGNWYWFDNSGEMATGWKKIADKWYYFNEEGAMKTGWVKYKDTWYYLDAKEGAMVSNAFIQSADGTGWYYLKPDGTLADKPDFTVEPEGLITVK

>5_SPN

MEINVSKLRTDLPQVGVQPYRQVHAHSTGNPHSTVQNEADYHWRKDPELGFFSHIVGNGCIMQVGPVDNGAWDVGGGWNAETYAAVELIESHSTKEEFMTDYRLYIELLRNLADEAGLPKTLDTGSLAGIKTHEYCTNNQPNNHSDHVDPYPYLAKWGISREQFKYDIENGLTIETGWQKNDTGYWYVHSDGSYPKDKFEKINGTWYYFDSSGYMLADRWRKHTDGNWYWFDNSGEMATGWKKIADKWYYFNEEGAMKTGWVKYKDTWYYLDAKEGAMVSNAFIQSADGTGWYYLKPDGTLADKPEFTVEPDGLITVK

>6_SPN

MEINVSKLRTDLPQVGVQPYRQVHAHSTGNPHSTVQNEADYHWRKDPELGFFSHIVGNGCIMQVGPVDNGAWDVGGGWNAETYAAVELIESHSTKEEFMTDYRLYIELLRNLADEAGLPKTLDTGSLAGIKTHEYCTNNQPNNHSDHVDPYPYLAKWGISREQFKHDIENGLTIETGWQKNDTGYWYVHSDGSYPKDKFEKINGTWYYFDSSGYMLADRWRKHTDGNWYWFDNSGEMATGWKKIAEKWYYFDVEGAMKTGWVKYKDTWYYLDAKEGAMVSNAFIQSADGTGWYYLKPDGTLADKPEFTVEPDGLITVK

>7_SPN

MEINVSKLRTDLPQVGVQPYRQVHAHSTGNPHSTVQNEADYHWRKDPELGFFSHIVGNGCIMQVGPVDNGAWDVGGGWNAETYAAVELIESHSTKEEFMTDYRLYIELLRNLADEAGLPKTLDTGSLAGIKTHEYCTNNQPNNHSDHVDPYPYLAKWGISREQFKYDIENGLTIETGWQKNDTGYWYVHSDGSYPKDKFEKINGTWYYFDSSGYMLADRWRKHTDGNWYWFDNSGEMATGWKKIAEKWYYFDVEGAMKTGWVKYKDTWYYLDAKEGAMVSNAFIQSADGTGWYYLKPDGTLADKPEFTVEPDGLITVK

>8_SPN GA13499

MEINVSKLRTDLPQVGVQPYRQVHAHSTGNSHSTVQNEADYHWRKDPELGFFSHIVGNGCIMQVGPVDNGAWDVGGGWNAETYAAVELIESHSTKEEFMTDYRLYIELLRNLADEAGLPKTLDTGSLAGIKTHEYCTNNQPNNHSDHVDPYPYLAKWGISREQFKHDIENGLTIETGWQKNDTGYWYVHSDGSYPKDKFEKINGTWYYFDSSGYMLADRWRKHTDGNWYWFDNSGEMATGWKKIADKWYYFNEEGAMKTGWVKYKDTWYYLDAKEGAMVSNAFIQSADGTGWYYLKPDGTLADKPEFTVEPDGLITVK

>9_SPN 801

MEINVSKLRTDLPQVGVQPYRQVHAHSTGNPHSTVQNEADYHWRKDPELGFFSHIVGNGCIMQVGPVDNGAWDVGGGWNAETYAAVELIESHSTKEEFMTDYRLYIELLRNLADEAGLPKTLDTGSLAGIKTHEYCTNNQPNNHSDHVDPYPYLAKWGISREQFKHDIENGLTIETGWQKNDTGYWYVHSDGSYPKDKFEKINGTWYYFDSSGYMLADRWRKHTDGNWYWFDNSGEMATGWKKIADKWYYFNEEGAMKTGWVKYKDTWYYLDAKEGAMVSNAFIQSADGTGWYYLKPDGTLADKPDFTVEPDGLITVK

>10_SPN PCS81218

MEINVSKLRTDLPQVGVQPYRQVHAHSTGNPHSTVQNEADYHWRKDPELGFFSHIVGNGCIMQVGPVDNGAWDVGGGWNAESYAAVELIESHSTKEEFMTDYRLYIELLRNLADEAGLPKTLDTGSLAGIKTHEYCTNNQPNNHSDHVDPYPYLAKWGISREQFKHDIENGLTIETGWQKNDTGYWYVHSDGSYPKDKFEKINGTWYYFDSSGYMLADRWRKHTDGNWYWFDNSGEMATGWKKIADKWYYFNEEGAMKTGWVKYKDTWYYLDAKEGAMVSNAFIQSADGTGWYYLKPDGTLADKPEFTVEPDGLITVK

>11_SPN 2081074

MEINVSKLRTDLPQVGVQPYRQVHAHSTGNPHSTVQNEADYHWRKDPELGFFSHIVGNGCIMQVGPVDNGAWDVGGGWNAETYAAVELIESHSTKEEFMTDYRLYIELLRNLADEAGLPKTLDTGSLAGIKTHEYCTNNQPNNHSDHVDPYPYLAKWGISREQFKYDIENDLTIETGWQKNDTGYWYVHSDGSYPKDKFEKINGTWYYFDSSGYMLADRWRKHTDGNWYWFDNSGEMATGWKKIADKWYYFNEEGAMKTGWVKYKDTWYYLDAKEGAMVSNAFIQSADGTGWYYLKPDGTLADKPEFTVEPDGLITVK

>12_SPN GA47597

MEINVSKLRTDLPQVGVQPYRQVHAHSTGNPHSTVQNEADYHCRKDPELGFFSHIVGNGCIMQVGPVDNGAWDVGGGWNAETYAAVELIESHSTKEEFMTDYRLYIELLRNLADEAGLPKTLDTGSLAGIKTHEYCTNNQPNNHSDHVDPYPYLAKWGISREQFKHDIENGLTIETGWQKNDTGYWYVHSDGSYPKDKFEKINGTWYYFDSSGYMLADRWRKHTDGNWYWFDNSGEMATGWKKIADKWYYFNEEGAMKTGWVKYKDTWYYLDAKEGAMVSNAFIQSADGTGWYYLKPDGTLADKPEFTVEPDGLITVK

>13_SPN 2082239

MEINVSKLRTDLPQVGVQPYRQVHAHSTGNPHSTVQNEADYHWRKDPELGFFSHIVGNGCIMQVGPVDNGAWDVGGGWNAETYAAVELIESHSTKEEFMTDYRLYIELLRNLADEAGLPKTLDTGSLAGIKTHEYCTNNQPNNHSDHVDPYPYLAKWGISREQFKYDIENGLTIETGWQKNDTGYWYVHSDGSYPKDKFEKINGTWYYFDSSGYMLADRWRKHTDGNWYWFDNSGEMATGWKKIADKWYYFDVEGAMKTGWVKYKDTWYYLDAKEGAMVSNAFIQSADGTGWYYLKPDGTLADKPEFTVEPDGLITVK

**Table S5**. Correspondence between *plyA* and PlyA alleles of *S. pneumoniae^a^*

| *plyA* allele (Fam_)*^b^* | PlyA allele*^c^* | Acc. No. of *plyA* alleles in databases | References | Specific activity (HU mg^−1^)*^d^* |
| --- | --- | --- | --- | --- |
| 1 (1) | 2 | AE005672 | ([Tettelin, et al. 2001](#_ENREF_7)) | 4.4 × 10^5^ |
| 2 (1) | 2 | GU968237 | ([Jefferies, et al. 2010](#_ENREF_4)) |  |
| 3 (1) | 2 | GU968219 | ([Jefferies, et al. 2010](#_ENREF_4)) |  |
| 4 (1) | 2 | FQ312045 | ([Donati, et al. 2010](#_ENREF_1)) |  |
| 5 (1) | 2 | GU968217 | ([Jefferies, et al. 2010](#_ENREF_4)) |  |
| 6 (1) | 2 | EF413941 | ([Jefferies, et al. 2007](#_ENREF_3)) |  |
| 7 (1) | 2 | CP000918 | Unpublished |  |
| 8 (1) | 2 | DQ251179 | ([Kirkham, et al. 2006](#_ENREF_5)) |  |
| 9 (1) | 2 | GU968385 | ([Jefferies, et al. 2010](#_ENREF_4)) |  |
| 10 (1) | 1 | GU968223 | ([Jefferies, et al. 2010](#_ENREF_4)) | 4.1 × 10^5^ |
| 11 (1) | 1 | EF413947 | ([Jefferies, et al. 2007](#_ENREF_3)) |  |
| 12 (1) | 1 | GU968317 | ([Jefferies, et al. 2010](#_ENREF_4)) |  |
| 13 (1) | 1 | CP002176 | Unpublished |  |
| 14 (2) | 5 | EF413960 | ([Jefferies, et al. 2007](#_ENREF_3)) | Non-hemolytic |
| 15 (1) | 1 | GU968247 | ([Jefferies, et al. 2010](#_ENREF_4)) |  |
| 16 (1) | 1 | − | − |  |
| 17 (1) | 1 | − | − |  |
| 18 (1) | 1 | CP000936 | Unpublished |  |
| 19 (1) | 2 | − | − |  |
| 20 (2) | 5 | EF368014 | ([Marks, et al. 2007](#_ENREF_6)) |  |
| 21 (2) | 10 | EF413934 | ([Jefferies, et al. 2007](#_ENREF_3)) | 1.1 × 10^5^ |
| 22 (1) | 11 | EF413933 | ([Jefferies, et al. 2007](#_ENREF_3)) | 5.9 × 10^5^ |
| 23 (1) | 2 | − | − |  |
| 24 (1) | 9 | GU968397 | ([Jefferies, et al. 2010](#_ENREF_4)) | 3.1 × 10^5^ |
| 25 (1) | 16 | GU968252 | ([Jefferies, et al. 2010](#_ENREF_4)) | Unknown |
| 26 (1) | 2 | GU968225 | ([Jefferies, et al. 2010](#_ENREF_4)) |  |
| 27 (1) | 2 | GU968238 | ([Jefferies, et al. 2010](#_ENREF_4)) |  |
| 28 (1) | 2 | GU968396 | ([Jefferies, et al. 2010](#_ENREF_4)) |  |
| 29 (2) | 3 | EF413957 | ([Jefferies, et al. 2007](#_ENREF_3)) | 6.9 × 10^3^ |
| 30 (1) | **20*^e^*** | − | − | Unknown |
| 31 (1) | 1 | − | − |  |
| 32 (1) | 2 | − | − |  |
| 33 (1) | 8 | GU968401 | ([Jefferies, et al. 2010](#_ENREF_4)) | 9.1 × 10^4^ |
| 34 (2) | 3 | − | − |  |
| 35 (1) | 1 | GU968221 | ([Jefferies, et al. 2010](#_ENREF_4)) |  |

*^a^* Novel *plyA* or PlyA alleles are shaded in a light or dark gray background respectively.

*^b^* Fam_1 and Fam_2 designate 1416- and 1410-bp-long *plyA_Spn_* alleles respectively.

*^c^* Numbering of PlyA alleles correspond to those previously proposed ([Jefferies, et al. 2010](#_ENREF_4)).

*^d^* The specific activities of the PlyA alleles [in hemolytic units (HU) per miligram of protein] were adapted from [Jefferies et al. (2007](#_ENREF_3)).

*^e^* Since up to 19 different PlyA alleles has been reported to date ([Jefferies, et al. 2007](#_ENREF_3); [Jefferies, et al. 2010](#_ENREF_4); [Harvey, et al. 2011](#_ENREF_2)), this new allele has preliminarily named as alelle 20.

**References**

Donati C, et al. 2010. Structure and dynamics of the pan-genome of *Streptococcus pneumoniae* and closely related species. Genome Biol. 11:R107.

Harvey RM, Ogunniyi AD, Chen AY, Paton JC. 2011. Pneumolysin with low hemolytic activity confers an early growth advantage to *Streptococcus pneumoniae* in the blood. Infect Immun. 79:4122−4130.

Jefferies JMC, et al. 2007. Presence of nonhemolytic pneumolysin in serotypes of *Streptococcus pneumoniae* associated with disease outbreaks. J Infect Dis. 196:936−944.

Jefferies JMC, et al. 2010. Identification of novel pneumolysin alleles from paediatric carriage isolates of *Streptococcus pneumoniae*. J Med Microbiol. 59:808−814.

Kirkham L-AS, et al. 2006. Identification of invasive serotype 1 pneumococcal isolates that express nonhemolytic pneumolysin. J Clin Microbiol. 44:151−159.

Marks M, et al. 2007. Influence of neutropenia on the course of serotype 8 pneumococcal pneumonia in mice. Infect Immun. 75:1586−1597.

Tettelin H, et al. 2001. Complete genome sequence of a virulent isolate of *Streptococcus pneumoniae*. Science 293:498−506.

**Nucleotide sequences of novel *plyA* alleles**

>*plyA*16

ATGGCAAATAAAGCAGTAAATGACTTTATACTAGCTATGAATTACGATAAAAAGAAACTCTTGACCCATCAGGGAGAAAGTATTGAAAATCGTTTCATCAAAGAGGGTAATCAGCTACCCGATGAGTTTGTTGTTATCGAAAGAAAGAAGCGGAGCTTGTCGACAAATACAAGTGATATTTCTGTAACAGCTACCAACGACAGTCGCCTCTATCCTGGAGCACTTCTCGTAGTGGATGAGACCTTGTTAGAGAATAATCCCACTCTTCTTGCGGTCGATCGTGCTCCGATGACTTATAGTATTGATTTGCCTGGTTTGGCAAGTAGCGATAGCTTTCTCCAAGTGGAAGACCCCAGCAATTCAAGTGTTCGCGGAGCGGTAAACGATTTGTTGGCTAAGTGGCATCAAGATTATGGTCAGGTCAATAATGTCCCAGCTAGAATGCAGTATGAAAAAATCACGGCTCACAGCATGGAACAACTCAAGGTCAAGTTTGGTTCTGACTTTGAAAAGACAGGGAATTCTCTTGATATTGATTTTAACTCTGTCCATTCAGGCGAAAAGCAGATTCAGATTGTTAATTTTAAGCAGATTTATTATACAGTCAGCGTAGACGCTGTTAAAAATCCAGGAGATGTGTTTCAAGATACTGTAACGGTAGAGGATTTAAAACAGAGAGGAATTTCTGCAGAGCGTCCTTTGGTCTATATTTCGAGTGTTGCTTATGGGCGCCAAGTCTATCTCAAGTTGGAAACCACGAGTAAGAGTGATGAAGTAGAGGCTGCTTTTGAAGCTTTGATAAAAGGAGTCAAGGTAGCTCCTCAGACAGAGTGGAAGCAGATTTTGGACAATACAGAAGTGAAGGCGGTTATTTTAGGGGGCGACCCAAGTTCGGGTGCCCGAGTTGTAACAGGCAAGGTGGATATGGTAGAGGACTTGATTCAAGAAGGCAGTCGCTTTACAGCAGATCATCCAGGCTTGCCGATTTCCTATACAACTTCTTTTTTACGTGACAATGTAGTTGCGACCTTTCAAAACAGTACAGACTATGTTGAGACTAAGGTTACAGCTTACAGAAACGGAGATTTACTGCTGGATCATAGTGGTGCCTATGTTGCCCAATATTATATTACTTGGGATGAATTATCCTATGATCATCAAGGTAAGGAAGTCTTGACTCCTAAGGCTTGGGACAGAAATGGGCAGGATTTGACGGCTCACTTTACCACTAGTATTCCTTTAAAAGGGAATGTTCGTAATCTCTCTGTCAAAATTAGAGAGTGTACCGGGCTTGCCTGGGAATGGTGGCGTACGGTTTATGAAAAAACCGATTTGCCACTAGTGCGTAAGCGGACGATTTCTATTTGGGGAACAACTCTCTATCCTCAGGTAGAGGATAAGGTAGAAAATGATTAG

>*plyA*17

ATGGCAAATAAAGCAGTAAATGACTTTATACTAGCTATGAATTACGATAAAAAGAAACTCTTGACCCATCAGGGAGAAAGTATTGAAAATCGTTTCATCAAAGAGGGTAATCAGCTACCCGATGAGTTTGTTGTTATCGAAAGAAAGAAGCGGAGCTTGTCGACAAATACAAGTGATATTTCTGTAACAGCTACCAACGACAGTCGCCTCTATCCTGGAGCACTTCTCGTAGTGGATGAGACCTTGTTAGAGAATAATCCCACTCTTCTTGCGGTCGATCGTGCTCCGATGACTTATAGTATTGATTTGCCTGGTTTGGCAAGTAGCGATAGCTTTCTCCAAGTGGAAGACCCCAGCAATTCAAGTGTTCGCGGAGCGGTAAACGATTTGTTGGCTAAGTGGCATCAAGATTATGGTCAGGTCAATAATGTCCCAGCTAGAATGCAGTATGAAAAAATCACGGCTCACAGCATGGAACAACTCAAGGTCAAGTTTGGTTCTGACTTTGAAAAGACAGGGAATTCTCTTGATATTGATTTTAACTCTGTCCATTCAGGCGAAAAGCAGATTCAGATTGTTAATTTTAAGCAGATTTATTATACAGTCAGCGTAGATGCTGTTAAAAATCCAGGAGATGTGTTTCAAGATACTGTAACGGTAGAGGATTTAAAACAGAGAGGAATTTCTGCAGAGCGTCCTTTGGTCTATATTTCGAGTGTTGCTTATGGGCGCCAAGTCTATCTCAAGTTGGAAACCACGAGTAAGAGTGATGAAGTAGAGGCTGCTTTTGAAGCTTTGATAAAAGGAGTCAAGGTAGCTCCTCAGACAGAGTGGAAGCAGATTTTGGACAATACAGAAGTGAAGGCGGTTATTTTAGGGGGCGATCCAAGTTCGGGTGCCCGAGTTGTAACAGGCAAGGTGGATATGGTAGAGGACTTGATTCAAGAAGGCAGTCGCTTTACAGCAGATCATCCAGGCTTGCCGATTTCCTATACAACTTCTTTTTTACGTGACAATGTAGTTGCGACCTTTCAAAACAGTACAGACTATGTTGAGACTAAGGTTACAGCTTACAGAAACGGAGATTTACTGCTGGATCATAGTGGTGCCTATGTTGCCCAATATTATATTACTTGGGATGAATTATCCTATGATCATCAAGGTAAGGAAGTCTTGACTCCTAAGGCTTGGGACAGAAATGGGCAGGATTTGACGGCTCACTTTACCACTAGTATTCCTTTAAAAGGGAATGTTCGTAATCTCTCTGTCAAAATTAGAGAGTGTACCGGGCTTGCCTGGGAATGGTGGCGTACGGTTTATGAAAAAACCGATTTGCCACTAGTGCGTAAGCGGACGATTTCTATTTGGGGAACAACTCTCTATCCTCAGGTAGAAGATAAGGTAGAAAATGACTAG

>*plyA*19

ATGGCAAATAAAGCAGTAAATGACTTTATACTAGCTATGAATTACGATAAAAAGAAACTCTTGACCCATCAGGGAGAAAGTATTGAAAATCGTTTCATCAAAGAGGGTAATCAGCTACCCGATGAGTTTGTTGTTATCGAAAGAAAGAAGCGGAGCTTGTCGACAAATACAAGTGATATTTCTGTAACAGCTACCAACGACAGTCGCCTCTATCCTGGAGCACTTCTCGTAGTGGATGAGACCTTGTTAGAGAATAATCCCACTCTTCTTGCGGTCGATCGTGCTCCGATGACTTATAGTATTGATTTGCCTGGTTTGGCAAGTAGCGATAGCTTTCTCCAAGTGGAAGACCCCAGCAATTCAAGTGTTCGCGGAGCGGTAAACGATTTGTTGGCTAAGTGGCATCAAGATTATGGTCAGGTCAATAATGTCCCAGCTAGAATGCAGTATGAAAAAATCACGGCTCACAGCATGGAACAACTCAAGGTCAAGTTTGGTTCTGACTTTGAAAAGACAGGGAATTCTCTTGATATTGATTTTAACTCTGTCCATTCAGGCGAAAAGCAGATTCAGATTGTTAATTTTAAGCAGATTTATTATACAGTCAGCGTAGACGCTGTTAAAAATCCAGGAGATGTGTTTCAAGATACTGTAACGGTAGAGGATTTAAAACAGAGAGGGATTTCTGCAGAGCGTCCTTTGGTCTATATTTCGAGTGTTGCTTATGGGCGCCAAGTCTATCTCAAGTTGGAAACCACGAGTAAGAGTGATGAAGTAGAGGCTGCTTTTGAAGCTTTGATAAAAGGAGTCAAGGTAGCTCCTCAGACAGAGTGGAAGCAGATTTTGGACAATACAGAAGTGAAGGCGGTTATTTTAGGGGGCGACCCAAGTTCGGGTGCCCGAGTTGTAACAGGCAAGGTGGATATGGTAGAGGACTTGATTCAAGAAGGCAGTCGCTTTACAGCAGATCATCCAGGCTTGCCGATTTCCTATACAACTTCTTTTTTACGTGACAATGTAGTTGCGACCTTTCAAAATAGTACAGACTATGTTGAGACTAAGGTTACAGCTTACAGAAACGGAGATTTACTGCTGGATCATAGTGGTGCCTATGTTGCCCAATATTATATTACTTGGAATGAATTATCCTATGATCATCAAGGTAAGGAAGTCTTGACTCCTAAGGCTTGGGACAGAAATGGGCAGGATTTAACGGCTCACTTTACCACTAGTATTCCTTTAAAAGGGAATGTTCGTAATCTCTCTGTCAAAATTAGAGAGTGTACCGGGCTTGCCTGGGAATGGTGGCGTACGGTTTATGAAAAAACCGATTTGCCACTAGTGCGTAAGCGGACGATTTCTATTTGGGGAACAACTCTCTATCCTCAGGTAGAAGATAAGGTAGAAAATGACTAG

>*plyA*23

ATGGCAAATAAAGCAGTAAATGACTTTATACTAGCTATGAATTACGATAAAAAGAAACTCTTGACCCATCAGGGAGAAAGTATTGAAAATCGTTTCATCAAAGAGGGTAATCAGCTACCCGATGAGTTTGTTGTTATCGAAAGAAAGAAGCGGAGCTTGTCGACAAATACAAGTGATATTTCTGTAACAGCTACCAACGACAGTCGCCTCTATCCTGGAGCACTTCTCGTAGTGGATGAGACCTTGTTAGAGAATAATCCCACTCTTCTTGCGGTCGATCGTGCTCCGATGACTTATAGTATTGATTTGCCTGGTTTGGCAAGTAGCGATAGCTTTCTCCAAGTGGAAGACCCCAGCAATTCAAGTGTTCGCGGAGCGGTAAACGATTTGTTGGCTAAGTGGCATCAAGATTATGGTCAGGTCAATAATGTCCCAGCTAGAATGCAGTATGAAAAAATCACGGCTCACAGCATGGAACAACTCAAGGTCAAGTTTGGTTCTGACTTTGAAAAGACAGGGAATTCTCTTGATATTGATTTTAACTCTGTCCATTCAGGCGAAAAGCAGATTCAGATTGTTAATTTTAAGCAGATTTATTATACAGTCAGCGTAGACGCTGTTAAAAATCCAGGAGATGTGTTTCAAGATACTGTAACGGTAGAGGATTTAAAACAGAGAGGAATTTCTGCAGAGCGTCCTTTGGTCTATATTTCGAGTGTTGCTTATGGGCGTCAAGTCTATCTCAAGTTGGAAACCACGAGTAAGAGTGATGAAGTAGAGGCTGCTTTTGAAGCTTTGATAAAAGGAGTCAAGGTAGCTCCTCAGACAGAGTGGAAGCAGATTTTGGACAATACAGAAGTGAAGGCGGTTATTTTAGGGGGCGACCCAAGTTCGGGTGCCCGAGTTGTAACAGGCAAGGTGGATATGGTAGAGGACTTGATTCAAGAAGGCAGTCGCTTTACAGCAGATCATCCAGGCTTGCCGATTTCCTATACAACTTCTTTTTTACGTGACAATGTAGTTGCGACCTTTCAAAATAGTACAGACTATGTTGAGACTAAGGTTACAGCTTACAGAAACGGAGATTTACTGCTGGATCATAGTGGTGCCTATGTTGCCCAATATTATATTACTTGGAATGAATTATCCTATGATCATCAAGGTAAGGAAGTCTTGACTCCTAAGGCTTGGGACAGAAATGGGCAGGATTTAACGGCTCACTTTACCACTAGTATTCCTTTAAAAGGGAATGTTCGTAATCTCTCTGTCAAAATTAGAGAGTGTACCGGGCTTGCCTGGGAATGGTGGCGTACGGTTTATGAAAAAACCGATTTGCCACTAGTGCGTAAGCGGACGATTTCTATTTGGGGAACAACTCTCTATCCTCAGGTAGAAGATAAGGTAGAAAATGACTAG

>*plyA*30

ATGGCAAATAAAGCAGTAAATGACTTTATACTAGCTATGAATTACGATAAAAAGAAACTCTTGACCCATCAGGGAGAAAGTATTGAAAATCGTTTCATCAAAGAGGGTAATCAGCTACCCGATGAGTTTGTTGTTATCGAAAGAAAGAAGCGGAGCTTGTCGACAAATACAAGTGATATTTCTGTAACAGCTACCAACGACAGTCGCCTCTATCCTGGAGCACTTCTCGTAGTGGATGAGACCTTGTTAGAGAATAATCCCACTCTTCTTGCGGTCGATCGTGCTCCGATGACTTATAGTATTGATTTGCCTGGTTTGGCAAGTAGCGATAGCTTTCTCCAAGTGGAAGACCCCAGCAATTCAAGTGTTCGCGGAGCGGTAAACGATTTGTTGGCTAAGTGGCATCAAGATTATGGTCAGGTCAATAATGTCCCAGCTAGAATGCAGTATGAAAAAATCACGGCTCACAGCATGGAACAACTCAAGGTCAAGTTTGGTTCTGACTTTGAAAAGACAGGGAATTCTCTTGATATTGATTTTAACTCTGTCCATTCAGGCGAAAAGCAGATTCAGATTGTTAATTTTAAGCAGATTTATTATACAGTCAGCGTAGACGCTGTTAAAAATCCAGGAGATGTGTTTCAAGATACTGTAACGGTAGAGGATTTAAAACAGAGAGGAATTTCTGCAGAGCGTCCTTTGGTCTATATTGCGAGTGTTGCTTATGGGCGCCAAGTCTATCTCAAGTTGGAAACCACGAGTAAGAGTGATGAAGTAGAGGCTGCTTTTGAAGCTTTGATAAAAGGAGTCAAGGTAGCTCCTCAGACAGAGTGGAAGCAGATTTTGGACAATACAGAAGTGAAGGCGGTTATTTTAGGGGGCGACCCAAGTTCGGGTGCCCGAGTTGTAACAGGCAAGGTGGATATGGTAGAGGACTTGATTCAAGAAGGCAGTCGCTTTACAGCAGATCATCCAGGCTTGCCGATTTCCTATACAACTTCTTTTTTACGTGACAATGTAGTTGCGACCTTTCAAAACAGTACAGACTATGTTGAGACTAAGGTTACAGCTTACAGAAACGGAGATTTACTGCTGGATCATAGTGGTGCCTATGTTGCCCAATATTATATTACTTGGGATGAATTATCCTATGATCATCAAGGTAAGGAAGTCTTGACTCCTAAGGCTTGGGACAGAAATGGGCAGGATTTGACGGCTCACTTTACCACTAGTATTCCTTTAAAAGGGAATGTTCGTAATCTCTCTGTCAAAATTAGAGAGTGTACCGGGCTTGCCTGGGAATGGTGGCGTACGGTTTATGAAAAAACCGATTTGCCACTAGTGCGTAAGCGGACGATTTCTATTTGGGGAACAACTCTCTATCCTCAGGTAGAGGATAAGGTAGAAAATGATTAG

>*plyA*31

ATGGCAAATAAAGCAGTAAATGACTTTATACTAGCTATGAATTACGATAAAAAGAAACTCTTGACCCATCAGGGAGAAAGTATTGAAAATCGTTTCATCAAAGAGGGTAATCAGCTACCCGATGAGTTTGTTGTTATCGAAAGAAAGAAGCGGAGCTTGTCGACAAATACAAGTGATATTTCTGTAACAGCTACCAACGACAGTCGCCTCTATCCTGGAGCACTTCTCGTAGTGGATGAGACCTTGTTAGAGAATAATCCCACTCTTCTTGCGGTCGATCGTGCTCCGATGACTTATAGTATTGATTTGCCTGGTTTGGCAAGTAGCGATAGCTTTCTCCAAGTGGAAGACCCCAGCAATTCAAGTGTTCGCGGAGCGGTAAACGATTTGTTGGCTAAGTGGCATCAAGATTATGGTCAGGTCAATAATGTCCCAGCTAGAATGCAGTATGAAAAAATCACGGCTCACAGCATGGAACAACTCAAGGTCAAGTTTGGTTCTGACTTTGAAAAGACAGGGAATTCTCTTGATATTGATTTTAACTCTGTCCATTCAGGCGAAAAGCAGATTCAGATTGTTAATTTTAAACAGATTTATTATACAGTCAGCGTAGACGCTGTTAAAAATCCAGGAGATGTGTTTCAAGATACTGTAACGGTAGAGGATTTAAAACAGAGAGGAATTTCTGCAGAGCGTCCTTTGGTCTATATTTCGAGTGTTGCTTATGGGCGCCAAGTCTATCTCAAGTTGGAAACCACGAGTAAGAGTGATGAAGTAGAGGCTGCTTTTGAAGCTTTGATAAAAGGAGTCAAGGTAGCTCCTCAGACAGAGTGGAAACAGATTTTGGACAATACAGAAGTGAAGGCGGTTATTTTAGGGGGCGACCCAAGTTCGGGTGCCCGAGTTGTAACAGGCAAGGTGGATATGGTAGAGGACTTGATTCAAGAAGGCAGTCGCTTTACAGCAGATCATCCAGGCTTGCCGATTTCCTATACAACTTCTTTTTTACGTGACAATGTAGTTGCGACCTTTCAAAACAGTACAGACTATGTTGAGACTAAGGTTACAGCTTACAGAAACGGAGATTTACTGCTGGATCATAGTGGTGCCTATGTTGCCCAATATTATATTACTTGGGATGAATTATCCTATGATCATCAAGGTAAGGAAGTCTTGACTCCTAAGGCTTGGGACAGAAATGGGCAGGATTTGACGGCTCACTTTACCACTAGTATTCCTTTAAAAGGGAATGTTCGTAATCTCTCTGTCAAAATTAGAGAGTGTACCGGGCTTGCCTGGGAATGGTGGCGTACGGTTTATGAAAAAACCGATTTGCCACTAGTGCGTAAGCGGACGATTTCTATTTGGGGAACAACTCTCTATCCTCAGGTAGAAGATAAGGTAGAAAATGACTAG

>*plyA*32

ATGGCAAATAAAGCAGTAAATGACTTTATACTAGCTATGAATTACGATAAAAAGAAACTCTTGACCCATCAGGGAGAAAGTATTGAAAATCGTTTCATCAAAGAGGGTAATCAGCTACCCGATGAGTTTGTTGTTATCGAAAGAAAGAAGCGGAGCTTGTCGACAAATACAAGTGATATTTCTGTAACAGCTACCAACGACAGTCGCCTCTATCCTGGAGCACTTCTCGTAGTGGATGAGACCTTGTTAGAGAATAATCCTACTCTTCTTGCGGTCGATCGTGCTCCGATGACTTATAGTATTGATTTGCCTGGTTTGGCAAGTAGCGATAGCTTTCTCCAAGTGGAAGACCCCAGCAATTCAAGTGTTCGCGGAGCGGTAAACGATTTGTTGGCTAAGTGGCATCAAGATTATGGTCAGGTCAATAATGTCCCAGCTAGAATGCAGTATGAAAAAATCACGGCTCACAGCATGGAACAACTCAAGGTCAAGTTTGGTTCTGACTTTGAAAAGACAGGGAATTCTCTTGATATTGATTTTAACTCTGTCCATTCAGGCGAAAAGCAGATTCAGATTGTTAATTTTAAGCAGATTTATTATACAGTCAGTGTAGATGCTGTTAAAAATCCAGGAGATGTGTTTCAAGATACTGTAACGGTAGAGGATTTAAAACAGAGAGGAATTTCTGCAGAGCGTCCTTTGGTCTATATTTCGAGTGTTGCTTATGGGCGCCAAGTCTATCTCAAGTTGGAAACCACGAGTAAGAGTGATGAAGTAGAGGCTGCTTTTGAAGCTTTGATAAAAGGAGTCAAGGTAGCTCCTCAGACAGAGTGGAAGCAGATTTTGGACAATACAGAAGTGAAGGCGGTTATTTTAGGGGGCGACCCAAGTTCGGGTGCCCGAGTTGTAACAGGCAAGGTGGATATGGTAGAGGACTTGATTCAAGAAGGCAGTCGCTTTACAGCAGATCATCCAGGCTTGCCGATTTCCTATACAACTTCTTTTTTACGTGACAATGTAGTTGCGACCTTTCAAAATAGTACAGACTATGTTGAAACTAAGGTTACAGCTTACAGAAACGGAGATTTACTGCTGGATCATAGTGGTGCCTATGTTGCCCAATATTATATTACTTGGAATGAATTATCCTATGATCATCAAGGTAAGGAAGTCTTGACTCCTAAGGCTTGGGACAGAAATGGGCAGGATTTAACGGCTCACTTTACCACTAGTATTCCTTTAAAAGGGAATGTTCGTAATCTCTCTGTCAAGATTAGAGAGTGTACCGGGCTTGCCTGGGAATGGTGGCGTACGGTTTATGAAAAAACCGATTTGCCACTAGTGCGTAAGCGGACGATTTCTATTTGGGGAACAACTCTCTATCCTCAGGTAGAAGATAAGGTAGAAAATGACTAG

>*plyA*34 (1410 nt)

ATGGCAAATAAAGCAGTAAATGACTTTATACTAGCTATGAATTACGATAAAAAGAAACTCTTGACCCATCAGGGAGAAAGTATTGAAAATCGTTTCATCAAAGAGGGTAATCAGCTACCCGATGAGTTTGTTGTTATCGAAAGAAAGAAGCGGAGCTTGTCGACAAATACAAGTGATATTTCTGTAACAGCTACCAACGACAGTCGCCTCTATCCTGGAGCACTTCTCGTAGTGGATGAGACCTTGTTAGAGAATAATCCCACTCTTCTTGCGGTCGATCGTGCTCCGATGACTTATAGTATTGATTTGCCTGGTTTGGCAAGTAGCGATAGCTTTCTCCAAGTGGAAGACCCCAGCAATTCAAGTGTTCGCGGAGCGGTAAACGATTTGTTGGCTAAGTGGCATCAAGATTATGGTCAGGTCAATAATGTCCCAGCTAGAATGCAGTATGAAAAAATCACGGCTCACAGCATGGAACAACTCAAGGTCAAGTTTGGTTCTGACTTTGAAAAGATAGGGAATTCTCTTGATATTGATTTTAACTCTGTCCATTCAGGAGAAAAGCAGATTCAGATTGTTAATTTTAAGCAGATTTATTATACAGTCAGCGTAGATGCTGTTAAAAATCCAGGAGATGTGTTTCAAGATACTGTAACGGTAGAGGATTTAAGGCAGAGAGGAATTTCTGCAGAGCGTCCTTTGGTCTATATTTCGAGTGTTGCTTATGGGCGCCAAGTCTATCTCAAGTTGGAAACCACGAGTAAGAGTGATGAAGTAGAGGCTGCTTTTGAATCTTTGATAAAAGGAGTAGCTCCTCAGACAGAGTGGAAGCAGATTTTGGACAATACAGAAGTGAAGGCGGTTATTTTAGGGGGCGACCCAAGTTCGGGTGCCCGAGTTGTAACAGGCAAGGTGGATATGGTAGAGGACTTGATTCAAGAAGGCAGTCGCTTTACAGCCGATCATCCAGGCTTGCCGATTTCCTATACAACTTCTTTTTTACGTGACAATGTAGTTGCGACCTTTCAAAACAGTACAGACTATGTTGAGACTAAGGTTACAGCTTACAGAAACGGAGATTTACTGCTGGATCATAGTGGTGCCTATGTTGCTCAATATTATATTACTTGGGATGAATTATCCTATGATCATCAAGGCAAGGAAGTCTTGACTCCTAAGGCTTGGGACAGAAATGGGCAGGATTTAACGGCTCACTTTACCACTAGTATTCCTTTAAAAGGGAATGTTCGTAATCTCTCTGTCAAAATTAGAGAGTGTACCGGGCTTGCCTGGGAATGGTGGCGTACGGTTTATGAAAAAACCGATTTGCCACTAGTGCGTAAGCGGACGATTTCTATTTGGGGAACAACTCTCTATCCTCAGGTAGAAGATAAGGTAGAAAATGACTAG

**Amino acid sequence of the novel PlyA*_Spn_*-20 allele**

>PlyA20

MANKAVNDFILAMNYDKKKLLTHQGESIENRFIKEGNQLPDEFVVIERKKRSLSTNTSDISVTATNDSRLYPGALLVVDETLLENNPTLLAVDRAPMTYSIDLPGLASSDSFLQVEDPSNSSVRGAVNDLLAKWHQDYGQVNNVPARMQYEKITAHSMEQLKVKFGSDFEKTGNSLDIDFNSVHSGEKQIQIVNFKQIYYTVSVDAVKNPGDVFQDTVTVEDLKQRGISAERPLVYIASVAYGRQVYLKLETTSKSDEVEAAFEALIKGVKVAPQTEWKQILDNTEVKAVILGGDPSSGARVVTGKVDMVEDLIQEGSRFTADHPGLPISYTTSFLRDNVVATFQNSTDYVETKVTAYRNGDLLLDHSGAYVAQYYITWDELSYDHQGKEVLTPKAWDRNGQDLTAHFTTSIPLKGNVRNLSVKIRECTGLAWEWWRTVYEKTDLPLVRKRTISIWGTTLYPQVEDKVEND

This novel allele differs from PlyA1 by a S238A conserved substitution.

**Fig. S1.** Diagram of SMG genes homologous to *S. pseudopneumoniae* SPPN_02090 (*llyA*1 and *llyA*2 subfamilies).

A. With the exception of this gene, *S. pneumoniae* and *S. pseudopneumoniae* genomes are syntenic in this region, whereas disparities are characteristic of *S. mitis* and *S. oralis* genomes. A SPPN_02090 ortholog is also present upstream of *pulA* in the *S. mitis* strains SK597 and SK1080.

B. A different subfamily of SPPN_02090 orthologous genes (*llyA*2) is located downstream of *parC* in many different *S. mitis* strains (also including SK597). Regions showing ≥90% sequence identity are represented by identical color and shading.

**Fig. S2.** Diagram of SMG genes (and gene products) homologous to *S. pseudopneumoniae* SPPN_04220 (*llyB* subfamily).

Thin arrows represent interrupted genes (pseudogenes). Regions showing ≥90% sequence identity are represented by identical color and/or shading.

***S. mitis***

B6 **cat**taggaatctccttttttcacattttaatctttcttattat**aacacaagttttttcga**

NCTC 12261 **cat**taggaatctccttttttcacattttaatctttcttattat**aacacaagttttttcga**

11/5 **cat**taggaatctccttttttcacattttaatctttcttattat**aacacaagttttttcga**

13/39 **cat**taggaatctccttttttcacattttaatctttcttattat**aacacaagttttttcga**

17/34 **cat**taggaatctccttttttcacattttaatctttcttattat**aacacaagttttttcga**

18/56 **cat**taggaatctccttttttcacattttaatctttcttattat**aacacaagttttttc**t**a**

29/42 **cat**taggaatctccttttttcacattttaatctttcttattat**aacacaagttttttcga**

F0392 **cat**taggaatctccttttttcacattttaatctttcttattat**aacacaagttttttcga**

SK95 **cat**taggaatctccttttttcacattttaatctttcttattat**aacacaagttttttcga**

SK321 **cat**taggaatctccttttttcacattttaatctttcttattat**aacacaagttttttcga**

SK569 **cat**taggaatctccttttttcacattttaatctttcttattat**aacacaagttttttcga**

SK575 **cat**taggaatctccttttttcacattttaatctttcttattat**aacacaagttttttcga**

SK579 **cat**taggaatctccttttttcacattttaatctttcttattat**aacacaagttttttcga**

SK616 **cat**taggaatctccttttttcacattttaatctttcttattat**aacacaagttttttcga**

***S. oralis***

Uo5 **cat**taggaatctccttttttcacattttaatctttcttattat**aacacaagttttttcga**

ATCC 35037 **cat**taggaatctccttttttcacattttaatctttcttattat**aacacaagttttttcga**

ATCC 49296 **cat**taggaatctccttttttcacattttaatctttcttattat**aacacaagttttttcga**

SK10 **cat**taggaatctccttttttcacattttaatctttcttattat**aacacaagttttttcga**

SK100 **cat**taggaatctccttttttcacattttaatctttcttattat**aacacaagttttttcga**

SK313 **cat**taggaatctccttttttcacattttaatctttcttattat**aacacaagttttttcga**

***S. infantis***

ATCC 700779 **cat**taggaatctccttttttcacattttaatctttcttattat**aacacaagt**c**t**ag**tcga**

X **cat**taggaatctccttttttcacattttaatctttcttattat**aacacaagt**c**t**ag**tcga**

***S. tigurinus***

AZ_3a **cat**taggaatctccttttttcacattttaatctttcttattat**aacacaagt**c**t**agct**ga**

1366 **cat**taggaatctccttttttcacattttaatctttcttattat**aacacaagttttttcga**

2425 **cat**taggaatctccttttttcacattttaatctttcttattat**aacacaagttttttcga**

2426 **cat**taggaatctccttttttcacattttaatctttcttattat**aacacaagttttttcga**

***Streptococcus* sp.**

F0407 **cat**taggaatctccttttttcacattttaatctttcttattat**aacacaagttttttcga**

GMD1S **cat**taggaatctccttttttcacattttaatctttcttattat**aacacaagttttttcga**

GMD2S **cat**taggaatctccttttttcacattttaatctttcttattat**aacacaagttttttcga**

GMD4S **cat**taggaatctccttttttcacattttaatctttcttattat**aacacaagttttttcga**

GMD6S **cat**taggaatctccttttttcacattttaatctttcttattat**aacacaagttttttcga**

SK643 **cat**taggaatctccttttttcacattttaatctttcttattat**aacacaagttttttcga**

SPAR10 **cat**taggaatctccttttttcacattttaatctttcttattat**aacacaagt**c**t**ag**tcga**

**************************************************** * *

B6 **ttttcactagaggaaatggattttatT**---------------------------------

NCTC 12261 **ttttcactagaggaaatggattttatT**---------------------------------

11/5 **ttttcactagaggaaatggattttatT**---------------------------------

13/39 **ttttcactagaggaaatggattttatT**---------------------------------

17/34 **ttttcactagaggaaat**a**gattttatT**---------------------------------

18/56 **ttttcactagaggaaatggattttatT**---------------------------------

29/42 **ttttcactagaggaaatggattttatT**---------------------------------

F0392 **ttttcactagaggaaatggatttt**g**tT**---------------------------------

SK95 **ttttcactagaggaaatggatttt**c**tT**---------------------------------

SK321 **ttttcactagaggaaat**a**gattttatT**---------------------------------

SK569 **ttttcactagaggaaatggattttatT**---------------------------------

SK575 **ttttcactagaggaaat**a**gattttatT**---------------------------------

SK579 **ttttcactagaggaaat**a**gattttatT**---------------------------------

SK616 **ttttcactagaggaaatggattttaT**----------------------------------

Uo5 **ttttcactagaggaaa**g**ggattttatt**ttatactcaatgaaaatcaaagagcaaactagg

ATCC 35037 **ttttcactagaggaaatggatttt**g**tT**---------------------------------

ATCC 49296 **ttttcactagaggaaatggattttatt**ttatactcaatgaaaatcaaagagcaaactagg

SK10 **ttttcactagaggaaatggattttatT**---------------------------------

SK100 **ttttcactagaggaaatggattttatT**---------------------------------

SK313 **ttttcactagaggaaatggatttt**c**tT**---------------------------------

ATCC 700779 **ttttcactagaggaaatggatttt**tc**tT**--------------------------------

X **ttttcactagaggaaatggatttt**tc**tT**--------------------------------

AZ_3a **ttttcactagaggaaatggatttt**c**tT**---------------------------------

1366 **ttttcactagaggaaatggatttt**c**tT**---------------------------------

2425 **ttttcactagaggaaatggatttt**c**tT**---------------------------------

2426 **ttttcactagaggaaatggatttt**c**tT**---------------------------------

F0407 **ttttcactagaggaaatggattttatT**---------------------------------

GMD1S **ttttcactagaggaaatggattttatT**---------------------------------

GMD2S **ttttcactagaggaaatggattttatT**---------------------------------

GMD4S **ttttcactagaggaaatggattttatT**---------------------------------

GMD6S **ttttcactagaggaaatggattttatT**---------------------------------

SK643 **ttttcactagaggaaa**c**ggattttatT**---------------------------------

SPAR10 **ttttcactagaggaaatggatttt**tc**tT**--------------------------------

**************** ******

B6 ------------------------------------------------------------

NCTC 12261 ------------------------------------------------------------

11/5 ------------------------------------------------------------

13/39 ------------------------------------------------------------

17/34 ------------------------------------------------------------

18/56 ------------------------------------------------------------

29/42 ------------------------------------------------------------

F0392 ------------------------------------------------------------

SK95 ------------------------------------------------------------

SK321 ------------------------------------------------------------

SK569 ------------------------------------------------------------

SK575 ------------------------------------------------------------

SK579 ------------------------------------------------------------

SK616 ------------------------------------------------------------

Uo5 aagctagccgcaggctg**tacttgagtacggcaagactaagctgacacggtttgaatttga**

ATCC 35037 ------------------------------------------------------------

ATCC 49296 aagctagccgcaggctg**tacttgagtacggcaaggctaagctgacacggtttgaatttga**

SK10 ------------------------------------------------------------

SK100 ------------------------------------------------------------

SK313 ------------------------------------------------------------

ATCC 700779 ------------------------------------------------------------

X ------------------------------------------------------------

AZ_3a ------------------------------------------------------------

1366 ------------------------------------------------------------

2425 ------------------------------------------------------------

2426 ------------------------------------------------------------

F0407 ------------------------------------------------------------

GMD1S ------------------------------------------------------------

GMD2S ------------------------------------------------------------

GMD4S ------------------------------------------------------------

GMD6S ------------------------------------------------------------

SPAR10 ------------------------------------------------------------

SK643 ------------------------------------------------------------

B6 --------------t**ag**c**aaat**a**ca**g**ctaggattgcacttt**t**gttgccaagat**a**gccttg**

NCTC 12261 --------------**cagtaaat**a**caactaggat**a**gcacttt**a**gttgc**t**aa**a**att**t**ccttg**

11/5 --------------**cagtaaat**a**caactaggat**a**gcacttt**a**gttgc**t**aa**a**att**t**ccttg**

13/39 --------------t**ag**c**aaat**a**ca**g**ctagga**ga**gcacttt**aa**ttgccaagat**g**g**a**cttg**

17/34 --------------**cagtaaatcaa**g**ctaggat**ga**cacttt**t**gttgccaagat**g**gccttg**

18/56 --------------t**ag**c**aaatcaa**g**ctaggat**g**g**t**acttt**t**g**c**t**a**ccaagatt**t**ccttg**

29/42 --------------**cagtaaat**a**caactaggat**a**gcacttt**a**gttgc**t**aa**a**att**t**ccttg**

F0392 --------------**cag**c**aaatcca**g**ctaggattg**tc**ctttgg**c**ttccaagatt**t**ccttg**

SK95 --------------tct**taaatcca**g**ctaggatt**atc**cttt**a**g**c**tgccaagattgccttg**

SK321 --------------**cagtaaatccaactaggat**a**gcactttggttgccaagat**ga**cctt**a

SK569 --------------**cagtaaatc**a**a**g**ctaggat**ga**cacttt**t**gttgccaagat**g**g**t**cttg**

SK575 --------------t**agtaaat**a**ca**g**ctaggattgcactttggtt**a**ccaagattgccttg**

SK579 --------------**cag**c**aaatc**g**a**g**cta**t**gat**ga**cagttt**t**gttgccaagat**g**gccttg**

SK616 --------------t**ag**c**aaat**a**ca**g**ctaggat**at**cactttggttgccaagatt**t**ccttg**

Uo5 **ttttcgaagagtat**t**ag**c**aaatcca**g**ctagga**g**tg**tc**cttt**a**g**ca**gccaaga**c**tgccttg**

ATCC 35037 --------------**cag**c**aaa**c**cca**g**ctag**t**attg**tc**ctttgg**c**tgccaagattgccttg**

ATCC 49296 **ttttcgaagagtat**t**ag**c**aaatcca**g**ctaggattg**tc**ctttgg**c**tgccaagattgccttg**

SK10 --------------**cag**c**aaatcca**g**ctaggattg**tc**ctttgg**c**tgccaagattgcctt**t

SK100 --------------**cag**c**aaatcca**g**ctaggattg**tc**cttt**t**gttgccaagattgccttg**

SK313 --------------tt**g**c**aaa**g**c**a**a**g**ctagg**c**tt**tgc**ct**cc**g**ac**tgcca**c**gatt**tt**cttg**

ATCC 700779 --------------tta**taaat**ga**a**g**ctaggat**c**g**tc**c**gcc**g**aca**gccaagatt**tt**cttg**

X --------------tta**taaat**ga**a**g**ctaggattg**tc**c**gcc**g**aca**gccaagatt**tt**cttg**

AZ_3a --------------tt**g**c**aaa**g**c**a**a**g**ctag**a**att**t**c**c**ct**cc**g**ac**tgcca**c**gatt**tt**cttg**

1366 --------------tt**g**c**aaa**g**c**a**a**g**ctaggatt**t**c**c**ct**cc**g**ac**tgcca**c**gatt**tt**cttg**

2425 --------------tt**g**c**aaa**g**c**a**a**g**ctaggatt**t**c**c**ct**cc**g**ac**tgcca**c**gatt**tt**cttg**

2426 --------------tt**g**c**aaa**g**c**a**a**g**ctaggatt**t**c**c**ct**cc**g**ac**tgcca**c**gatt**tt**cttg**

F0407 --------------**cag**c**aaatcca**g**ctaggattg**tc**ctttgg**c**tgccaagattgccttg**

GMD1S --------------**cag**c**aaatcca**g**ctaggattg**tc**ctttgg**c**tgccaagattgccttg**

GMD2S --------------**cag**c**aaatcca**g**ctaggattg**tc**ctttgg**c**tgccaagattgccttg**

GMD4S --------------**cag**c**aaatcca**g**ctaggattg**tc**ctttgg**c**tgccaagattgccttg**

GMD6S --------------**cag**c**aaatcca**g**ctaggattg**tc**ctttgg**c**tgccaagattgccttg**

SPAR10 --------------tta**taaat**ga**a**g**ctaggattg**tc**c**gcc**g**aca**gccaagatt**tt**cttg**

SK643 --------------**cag**c**aaatc**g**a**g**cta**t**gat**ga**ca**c**ttt**t**gttgccaagat**g**gccttg**

*** * *** * * * ***

B6 **ccttcttttatcaa**agggtgacgaaacagtgagaaatacagttggatagtcatggcaacc

NCTC 12261 **ccttcttttatcaa**ggggtgacgaaacagtgagaaatacagttgaatggtcatggcaacc

11/5 **ccttcttttatcaa**ggggtgacgaaacagtgagaaatacagttgaatggtcatggcaacc

13/39 **ccttcttttatcaa**ggggtgacggaaaagcgaaaagtacagttgaatggtcatggcaacc

17/34 **cc**a**tcttttatcaa**aggatgacggaagagtgagaagtacaattgaatggtcatggcaacc

18/56 **ccttcttttatcaa**agggtgacgaaacagtgaaaaataaagttgtatggtcatggcaacc

29/42 **ccttcttttatcaa**ggggtgacgaaacagtgagaaatacagttgaatggtcatggcaacc

F0392 **ccttcttttatca**ggggatggcggaaaagtgaaaagtacagttggatagtcatggcaacc

SK95 **ccttcttttatca**ggggatggcggaaaagtgaaaaatacagttgggtggtcatggcaacc

SK321 **ccttcttttatcaa**ggggtgacgaaacagtgagaaatacagttggatggtcatggcaacc

SK569 **ccttcttttatcaa**ggggtggcgaaacagtgagaagtacagctgaatagtcatggcaacc

SK575 **ccttcttttatcaa**ggggtgacggaagagtgagaagtagagttgaatggtcatggcaacc

SK579 **ccttcttttatcaa**gggatgacggaacagtgaaaagtacagttgaatggtcatggcaacc

SK616 **ccttcttttatcaa**ggggtggcggaaaagcgaaaagtacagttggatggtcatggcaacc

Uo5 **ccttcttttatca**ggggatggcggaaaagtgagaagtacagttgggtggtcatggcaacc

ATCC 35037 **ccttcttttatca**ggggatggcggaaaagtgaaaagtacagttgtgtagtcatggcaacc

ATCC 49296 **ccttcttttatca**gggggtgacggaaaagtgagaagtacagttgggtggtcatggcgacc

SK10 **ccttcttttatca**ggggatggcggaaaagtgaaaagtacagttgggtagtcatggcaacc

SK100 **ccttcttttatca**ggggatggcggaaaagtgaaaagtacagttgggtagtcatggcaacc

SK313 **ccttcttttatc**c**a**agggtgtcgggaaagggagaagtaaagttgggcagtcatcacaacc

ATCC 700779 **ccttcttttatc**c**a**agggtgttgagagagtgagatataaagctgtgcagtcataacaagc

X **ccttcttttatc**c**a**agggtgctgagagagtgagatataaagctgtgcagtcataacaagc

AZ_3a **ccttcttttatc**c**a**agggtgtcgggaaagggagaagtaaagttggacagtcatcacaacc

1366 **ccttcttttatc**c**a**agggtgtcgggaaagggagaagtaaagttggacagtcatcacaacc

2425 **ccttcttttatc**c**a**agggtgtcgggaaagggagaagtaaagttggacagtcatcacaacc

2426 **ccttcttttatc**c**a**agggtgtcgggaaagggagaagtaaagttggacagtcatcacaacc

F0407 **ccttcttttatca**ggggatgacggaaaagtgaaaagtacagttgggtggtcatggcaacc

GMD1S **ccttcttttatca**ggggatggcggaaaagtgaaaagtacagttgggtggtcatggcaacc

GMD2S **ccttcttttatca**ggggatggcggaaaagtgaaaagtacagttgggtggtcatggcaacc

GMD4S **ccttcttttatca**ggggatggcggaaaagtgaaaagtacagttgggtggtcatggcaacc

GMD6S **ccttcttttatca**ggggatggcggaaaagtgaaaagtacagttgggtggtcatggcaacc

SK643 **ccttcttttatcaa**gggatgacggaacagtgagaaataaagttggatggtcatggcaacc

SPAR10 **ccttcttttatc**c**a**aggatgctgagagagtgagatataaagctgtgcagtcataacaagc

** ********* ** ** * * ** ** * ** * ** ***** * * *

**Fig. S3.** Multiple alignment of the DNA region located between the 3’ end of *dinF* and the gene located immediately downstream in selected SMG lacking a *lytA*_SMG_ gene.

The orientation of the sequences corresponds to that in the genome of the *S. pneumoniae* D39 strain. The nucleotides that match those of the *pl*REP copy overlapping the 3’ end of *dinF* are shown in a black background. Asterisks indicate identical nucleotides in all the sequences. Hyphens indicate gaps introduced to maximize similarity. The termination codon of *dinF* is marked in a red background whereas the initiation codon of the downstream gene is highlighted in a green background. In *S. oralis* strains Uo5 and ATCC 49296, an intervening BOX element is present (boxC is labeled in blue; boxA is boldfaced and labeled in gray).

**Fig. S4.** Diagram of the approximate location and orientation of genes *cinA, recA,* and *dinF* in the chromosomes of different streptococci.

Only complete genomes were analyzed. The first gene of each genome is *dnaA*. The positions were calculated as the percentage of the respective genome. Interestingly, *S. thermophilus* strains lack a functional *dinF* gene. Note that in *S. salivarius*, the location and orientation of the three genes appear to be strain-specific. *Ssa*, *S. salivarius*; *Sgl*, *S. gallolyticus*; *Sth*, *S. thermophilus*; *Ssu,* *S. suis*; *Smu*, *S. mutans*.

**Fig. S5.** Chromosomal rearrangement in the serotype 3 equine *S. pneumoniae* A45 strain.

The genome of the D39 strain is shown for comparison. The *plyA* gene and the capsular cluster (*cap/cps*) are shown in light and dark blue respectively. Several genes encoding choline-binding proteins are shown as red arrows that indicate the direction of transcription. Genes are identified as ordered in D39 (prefix SPD_)/A45 (SPNA45_) and correspond to the following: 0126/01912 (*pspA*); 0345/01670 (*cbpC*); 0357/01659 (*cbpF*); 0821/01231 (*cbpE*); 0853/01263 (*lytB*); 1403/00649 (*lytC*); 1726/− (*plyA*); 1737/− (*lytA_Spn_*); 1965/00072 (*pcpA*); 2017/02044 (*cbpA*); 2028/02054 (*cbpD*). The open reading frames SPNA45_01659 and SPNA45_01670 appear to correspond to pseudogenes. The chromosomal fragments of strain A45 that are inverted with respect to D39 are labeled in red. The chromosomal fragment that has been translocated (but not inverted) is marked in yellow. Hatched and blue boxes correspond to complete or defective prophages respectively.

**Fig. S6.** Alignment of the 8.6 kb DNA fragment found in the *plyA*−*lytA* island of various pneumococcal isolates with the sequences deposited in the databases.

For complete genomes, the accession no. and nucleotide positions are indicated in parentheses. The inset shows the percentages of nucleotide similarity compared with the fragment of strain GA43257. Predicted proteins were compared with the Pfam database: PF03009, *GDPD* (glycerophosphoryl diester phosphodiesterase family); PF13343, *SBP_bac_6* (bacterial extracellular solute-binding protein); PF00005, *ABC_tran* (ATP-binding domain of ABC transporters); PF08402, *TOBE_2* (TOBE domain); PF00528, *BPD_transp_1* (binding-protein-dependent transport system inner membrane component); PF02308, *MgtC* (MgtC family); PF13419, *HAD_2* (haloacid dehalogenase-like hydrolase); PF00356, *LacI* (bacterial regulatory proteins, lacI family); PF13416, *SBP_bac_8* (bacterial extracellular solute-binding protein).

**Fig. S7.** Alignment of the putative, defective prophage found in the *plyA*−*lytA* island of various pneumococcal isolates with the sequences deposited in the databases.

Potential phage genes are also shown as arrows indicating the direction of transcription. Blackened arrows account for genes encoding hypothetical proteins. The inset shows the percentages of nucleotide similarity compared with the defective prophage of strain GA02270. Predicted proteins were compared with the Pfam database: PF01695, *IstB_IS21* (IstB-like ATP binding protein); PF09524, *Phg_2220_C* (Conserved phage C-terminus); PF09681*, Phage_rep_org_N* (N-terminal phage replisome organiser); PF09669, *Phage_pRha* (Phage regulatory protein Rha); PF01381, *HTH_3* (Helix-turn-helix); PF00717, *Peptidase_S24* (Peptidase S24-like); PF13443, *HTH_26* (Helix-turn-helix); PF00589, *Phage_integrase* (Phage integrase family); PF14659, *Phage_int_SAM_3* (Phage integrase, N-terminal SAM-like domain).

**Fig. S8.** Biofilm formation capacity of *lytA, plyA,* and *plyA lytA* mutants of the *S. pneumoniae* strain R6.

The non-encapsulated R6 strain was used as a control. Cells were grown in C medium for 6 h at 34°C. Open and filled bars indicate growth and biofilm formation, respectively. In all panels, the results represent the means ± standard errors from at least four independent experiments, each performed in triplicate. *, *P* <0.05 (compared to the R6 or R924 strains; ANOVA with *post-hoc* tests).

**Fig. S9.** Dendrogram of *S. pneumoniae* genomes showing the arrangement distribution of the *plyA*—*lytA* island.

The number of additional genomes represented in each branch is shown in parentheses. Red diamonds indicate the location of the 44 *S. pneumoniae* genomes analyzed by Donati et al. (2010). Group II non-Ec-*Spn* are indicated in a gray background. The figure was slightly modified from <http://www.ncbi.nlm.nih.gov/genome/?term=CP000410> ([Tatusova, et al. 2014](#_ENREF_22)). The names of the different arrangements (A—H) are as in Fig. 6.

**References**

Andrewes FW, Horder TJ 1906. A study of the streptococci pathogenic for man. Lancet. 168:708−713.

Arbique JC, et al. 2004. Accuracy of phenotypic and genotypic testing for identification of *Streptococcus pneumoniae* and description of *Streptococcus pseudopneumoniae* sp. nov. J Clin Microbiol. 42:4686−4696.

Bridge PD, Sneath PHA 1982. *Streptococcus gallinarum* sp. nov. and *Streptococcus oralis* sp. nov. Int J Syst Bacteriol. 32:410−415.

Camelo-Castillo A, Benítez-Páez A, Belda-Ferre P, Cabrera-Rubio R, Mira A 2014. *Streptococcus dentisani* sp. nov., a new member of the mitis group. Int J Syst Evol Microbiol. 64:60−65.

Chester FD. 1901. A manual of determinative bacteriology. New York: The MacMillan Co.

Donati C, et al. 2010. Structure and dynamics of the pan-genome of *Streptococcus pneumoniae* and closely related species. Genome Biol. 11:R107.

Glazunova OO, Raoult D, Roux V 2006. *Streptococcus massiliensis* sp. nov., isolated from a patient blood culture. Int J Syst Evol Microbiol. 56:1127−1131.

Hanahan D 1983. Studies on transformation of *E. coli* with plasmids. J Mol Biol. 166:557−580.

Handley P, Coykendall A, Beighton D, Hardie JM, Whiley RA 1991. *Streptococcus crista* sp. nov., a viridans streptococcus with tufted fibrils, isolated from the human oral cavity and throat. Int J Syst Bacteriol. 41:543−547.

Huch M, et al. 2013. *Streptococcus rubneri* sp. nov., isolated from the human throat. Int J Syst Evol Microbiol. 63:4026−4032.

Kawamura Y, et al. 1998. *Streptococcus peroris* sp. nov. and *Streptococcus infantis* sp. nov., new members of the *Streptococcus mitis* group, isolated from human clinical specimens. Int J Syst Bacteriol. 48:921−927.

Kilian M, Mikkelsen L, Henrichsen J 1989. Taxonomic study of viridans streptococci: description of *Streptococcus gordonii* sp. nov. and emended descriptions of *Streptococcus sanguis* (White and Niven 1946), *Streptococcus oralis* (Bridge and Sneath 1982), and *Streptococcus mitis* (Andrewes and Horder 1906). Int J Syst Bacteriol. 39:471−484.

Lacks S, Hotchkiss RD 1960. A study of the genetic material determining an enzyme activity in *Pneumococcus*. Biochim Biophys Acta. 39:508−518.

Martín V, Mañes-Lázaro R, Rodríguez JM, Maldonado-Barragán A 2011. *Streptococcus lactarius* sp. nov., isolated from breast milk of healthy women. Int J Syst Evol Microbiol. 61:1048−1052.

Moscoso M, Claverys JP 2004. Release of DNA into the medium by competent *Streptococcus pneumoniae*: kinetics, mechanism and stability of the liberated DNA. Mol Microbiol. 54:783−794.

Moscoso M, Domenech M, García E 2010. Vancomycin tolerance in clinical and laboratory *Streptococcus pneumoniae* isolates depends on reduced enzyme activity of the major LytA autolysin or cooperation between CiaH histidine kinase and capsular polysaccharide. Mol Microbiol. 77:1052−1064.

Moscoso M, García E, López R 2006. Biofilm formation by *Streptococcus pneumoniae*: role of choline, extracellular DNA, and capsular polysaccharide in microbial accretion. J Bacteriol. 188:7785−7795.

Mosser JL, Tomasz A 1970. Choline-containing teichoic acid as a structural component of pneumococcal cell wall and its role in sensitivity to lysis by an autolytic enzyme. J Biol Chem. 245:287−298.

Romero P, López R, García E 2004. Characterization of LytA-like *N*-acetylmuramoyl-L-alanine amidases from two new *Streptococcus mitis* bacteriophages provides insights into the properties of the major pneumococcal autolysin. J Bacteriol. 186:8229−8239.

Sambrook J, Russell DW. 2001. Molecular Cloning. A Laboratory Manual. Cold Spring Harbor, New York: Cold Spring Harbor Laboratory Press.

Sánchez-Puelles JM, Sanz JM, García JL, García E 1992. Immobilization and single-step purification of fusion proteins using DEAE-cellulose. Eur J Biochem. 203:153−159.

Tabor S. 1990. Expression using the T7 RNA polymerase/promoter system. In: Ausubel FM, et al., editors. Current protocols in molecular biology. New York: Green Publishing Associates Inc. and John Wiley & Sons. p. 16.12.11−16.12.11.

Tatusova T, Ciufo S, Fedorov B, O'Neill K, Tolstoy I 2014. RefSeq microbial genomes database: new representation and annotation strategy. Nucleic Acids Res. 42:D553−D559.

Tong H, Gao X, Dong X 2003. *Streptococcus oligofermentans* sp. nov., a novel oral isolate from caries-free humans. Int J Syst Evol Microbiol. 53:1101−1104.

Usobiaga P, et al. 1996. Structural organization of the major autolysin from *Streptococcus pneumoniae*. J Biol Chem. 271:6832−6838.

Whiley RA, et al. 1990. *Streptococcus parasanguis* sp. nov., an atypical viridans *Streptococcus* from human clinical specimens. FEMS Microbiol Lett. 68:115−122.

White JC, Niven CF, Jr. 1946. *Streptococcus* s.b.e.: a streptococcus associated with subacute bacterial endocarditis. J Bacteriol. 51:717−722.

Willcox MD, Zhu H, Knox KW 2001. *Streptococcus australis* sp. nov., a novel oral streptococcus. Int J Syst Evol Microbiol. 51:1277−1281.

Woo PCY, et al. 2002. *Streptococcus sinensis* sp. nov., a novel species isolated from a patient with infective endocarditis. J Clin Microbiol. 40:805−810.

Zbinden A, et al. 2012. *Streptococcus tigurinus* sp. nov., isolated from blood of patients with endocarditis, meningitis and spondylodiscitis. Int J Syst Evol Microbiol. 62:2941−2945.

Zhang M, et al. 2013. Isolation and characterization of *Streptococcus troglodytidis* sp. nov., from a foot abscess of a chimpanzee (*Pan troglodytes*). Int J Syst Evol Microbiol. 63:449−453.
